# A Mitochondria-Targeting Fluorescent Probe for the Dual Sensing of Hypochlorite and Viscosity without Signal Crosstalk in Living Cells and Zebrafish

**DOI:** 10.3390/molecules29133059

**Published:** 2024-06-27

**Authors:** Chao Gao, Dan-Dan Chen, Lin Zhang, Ming-Lan Ma, Hu-Wei Liu, Hai-Rong Cui

**Affiliations:** Synergy Innovation Centre of Biological Peptide Antidiabetics of Hubei Province, College of Life Science, Wuchang University of Technology, Wuhan 430223, China; gc110312@126.com (C.G.); 13545051557@163.com (D.-D.C.); hbhghaly@126.com (L.Z.); yuebianxing82@163.com (M.-L.M.); hwliu@pku.edu.cn (H.-W.L.)

**Keywords:** hypochlorite, viscosity, bioimaging

## Abstract

Hypochlorite (ClO^−^) and viscosity both affect the physiological state of mitochondria, and their abnormal levels are closely related to many common diseases. Therefore, it is vitally important to develop mitochondria-targeting fluorescent probes for the dual sensing of ClO^−^ and viscosity. Herein, we have explored a new fluorescent probe, **XTAP**–**Bn**, which responds sensitively to ClO^−^ and viscosity with off–on fluorescence changes at 558 and 765 nm, respectively. Because the emission wavelength gap is more than 200 nm, **XTAP**–**Bn** can effectively eliminate the signal crosstalk during the simultaneous detection of ClO^−^ and viscosity. In addition, **XTAP**–**Bn** has several advantages, including high selectivity, rapid response, good water solubility, low cytotoxicity, and excellent mitochondrial-targeting ability. More importantly, probe **XTAP**–**Bn** is successfully employed to monitor the dynamic change in ClO^−^ and viscosity levels in the mitochondria of living cells and zebrafish. This study not only provides a reliable tool for identifying mitochondrial dysfunction but also offers a potential approach for the early diagnosis of mitochondrial-related diseases.

## 1. Introduction

Mitochondria, as essential energy-supplying organelles, play crucial roles in many cellular processes, including central metabolism, signal transduction, and cell apoptosis [1]. In cellular systems, mitochondrial viscosity is regarded as a crucial parameter for assessing mitochondrial status because it is closely associated with the mitochondrial respiratory state and mitochondrial functions [2]. Nevertheless, abnormal viscosity expression in mitochondria has been associated with many diseases. For example, aberrant mitochondrial viscosity will lead to mitochondrial damage, which induces the production of inflammatory cytokines, thereby activating inflammation disease [3]. In addition, the abnormal viscosity of mitochondria can impede mitochondrial metabolism, which in turn affects the metabolism and accumulation of fat in liver cells, consequently causing the fatty liver [4]. Furthermore, it has been found that the aberrant mitochondrial viscosity may cause mitochondrial dysfunction, which increases intracellular oxidative stress and promotes neuronal death, ultimately inducing Parkinson’s disease [5]. Meanwhile, hypochlorite (ClO^−^), as a highly reactive oxygen species (ROS), is mainly produced in mitochondria by the peroxidation of hydrogen peroxide (H_2_O_2_) with chloride ions (Cl^−^) under the catalysis of myeloperoxidase (MPO) [6]. Mitochondrial ClO^−^ plays a key role in fighting against external pathogens and regulating redox homeostasis [7]. However, excessive ClO^−^ levels can lead to oxidative stress and DNA damage, which cause some inflammatory diseases, cystic fibrosis, diabetes, and neurodegenerative disease [8,9]. It is worth noting that during oxidative stress stimulation, ClO diffusion is closely related to cellular viscosity [9]. Therefore, the simultaneous detection of ClO^−^ and viscosity in mitochondria will be particularly useful for the corresponding biological research and the clinical diagnosis of diseases.

**Scheme 1:**
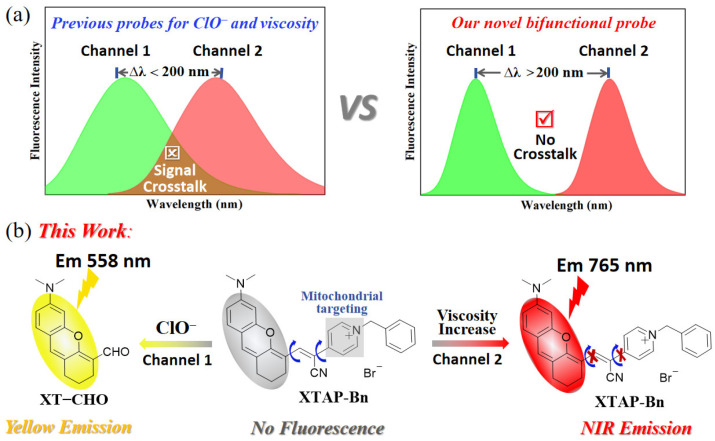
(**a**) Illustration of a dual-response fluorescent probe with signal crosstalk and without signal crosstalk. (**b**) The proposed mechanism of probe **XTAP**–**Bn** toward ClO^−^ and viscosity.

Fluorescence imaging technology has become a powerful tool for biological system monitoring, mainly due to its advantages of simple operation, high spatiotemporal resolution, and noninvasive detection [10]. Recently, numerous fluorescent probes have been studied for the independent detection of ClO^−^ [11,12,13,14,15,16,17,18] (Table S1) or viscosity [19,20,21,22,23,24,25] (Table S2). However, many of these probes were severely hydrophobic, requiring the use of a large amount of toxic organic co-solvents [11,12,14,15,17,18]. Some other probes lacked the mitochondrial-targeting function [11,12,13,14,17,18,19,20,22,24] and needed a long response time (several minutes) [11,13,16,17,18]. Obviously, the above problems seriously hinder the biological applicability of these studied probes. Furthermore, the fluorescent probes for the simultaneous detection of ClO^−^ and viscosity have rarely been investigated [26,27,28,29] (Table S3). Bifunctional fluorescent probes could shorten the detection time, reduce tool synthesis costs, and simplify testing procedures. More importantly, bifunctional fluorescent probes allow one to effectively avoid the fluorescence interference caused by the combination of two different probes in complex physiological environments [30]. Unfortunately, the reported bifunctional probes for ClO^−^ and viscosity generally suffer from the problem of signal crosstalk during detection due to the insufficient difference in emission wavelength, resulting in false positive signals and erroneous judgment (Scheme 1a). To overcome signal crosstalk, the fluorescence wavelength difference (Δλ) between two channels of an ideal bifunctional probe should be larger than 200 nm, which is twice the half-width of a regular fluorescence peak [31]. Considering all the above, it is vitally important to explore a water-soluble fluorescent probe for the fast and simultaneous detection of ClO^−^ and viscosity in mitochondria without signal crosstalk.

Herein, we explore a novel bifunctional fluorescent probe, **XTAP**–**Bn**, which can monitor ClO^−^ and viscosity in two emission channels without signal crosstalk (Scheme 1b). The probe **XTAP**–**Bn** was designed based on a typical D–π–A structure, in which the C=C bond (π linker) bridges the xanthene (**XT**) skeleton (electron donor, D) and the acrylonitrile–pyridinium (**AP**) moiety (electron acceptor, A). **XTAP**–**Bn**’s D–π–A structure provides the intramolecular rotors, which cause fluorescence quenching in PBS. While increasing viscosity, the intramolecular rotation of **XTAP**–**Bn** is inhibited, resulting in a bright NIR emission (λ_em_ = 765 nm). Meanwhile, the C=C bond of **XTAP**–**Bn** can be specifically oxidized by ClO^−^, which produces the fluorophore **XT**–**CHO** and releases an intense yellow emission (λ_em_ = 558 nm). Given the huge wavelength gap (Δλ = 207 nm), **XTAP**–**Bn** can effectively eliminate the signal crosstalk during the simultaneous detection of ClO^−^ and viscosity. In addition, **XTAP**–**Bn** responded to ClO^−^ rapidly (within 12 s) and selectively, and exhibited high sensitivity to viscosity. Owing to its excellent mitochondria-targeting ability, **XTAP**–**Bn** can simultaneously detect ClO^−^ and viscosity in the mitochondria of living cells. More importantly, it has been successfully employed to monitor the dynamic change of ClO^−^ and viscosity levels in zebrafish, indicating its excellent applicability in vivo.

## 2. Results and Discussion

### 2.1. Synthesis and Characterization of Probe XTAP–Bn

**Scheme 2:**
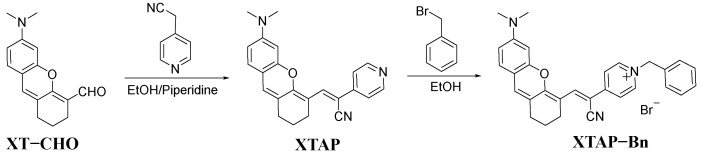
Synthetic route of probe **XTAP**–**Bn**.

In brief, the probe **XTAP**–**Bn** was synthesized using a two-step reaction (Scheme 2). Initially, the Knoevenagel condensation reaction of compound **XT**–**CHO** and 2-(pyridin-4-yl)acetonitrile produced the intermediate **XTAP**. Subsequently, the following quaternization reaction of **XTAP** with (bromomethyl)benzene produced **XTAP**–**Bn** with a 74% yield. The structures of the above compounds were confirmed with ^1^H NMR, ^13^C NMR, and HRMS, which can be found in the Supporting Information (Figures S10–S15).

### 2.2. Spectroscopic Response to Viscosity

**Figure 1:**
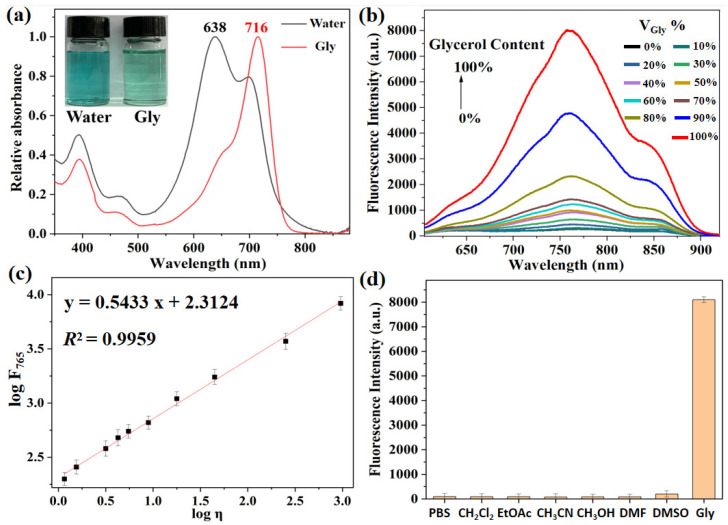
(**a**) Absorption spectra of **XTAP**–**Bn** (5 µM) in water and glycerol, respectively. Insert: the corresponding photos taken under sunlight. (**b**) The fluorescence spectra of **XTAP**–**Bn** (5 µM) in different ratios of a water–glycerol mixture (glycerol from 0 to 100%). λ_ex_ = 620 nm. (**c**) The linear relationship between logF_765_ and logη in the water–glycerol mixture. (**d**) The fluorescence intensity of **XTAP**–**Bn** (5 µM) at 765 nm in different solvents. Excitation was at the maximum absorption wavelength of each one.

To accurately reveal the viscosity-sensitive behaviors of **XTAP**–**Bn**, its optical properties were investigated in water–glycerol mixtures with different glycerol contents (V_Gly_ %). Initially, the UV-absorption spectra and fluorescence emission spectra of **XTAP**–**Bn** (5 µM) in pure glycerol and water were studied. As shown in Figure 1a, the maximum absorption peak of **XTAP**–**Bn** in pure water was at 638 nm. When using glycerol with high viscosity as a solvent, the maximum absorption peak of **XTAP**–**Bn** was red-shifted to 716 nm. This is because, in a high-viscosity glycerol medium, the molecular rotation of **XTAP**–**Bn** was restricted, and the molecular conjugation was increased, thereby causing an obvious red-shift in the absorption wavelength [23]. Moreover, it was learned from the fluorescence spectra that **XTAP**–**Bn** was almost non-fluorescent in pure water (Figure 1b). However, the fluorescence intensity at 765 nm (F_765_) was enhanced almost 105-fold, with increasing viscosity (η) from 0.89 cP (H_2_O, 25 °C) to 945 cP (Glycerol, 25 °C). In a low-viscosity environment, the rotation of **XTAP**–**Bn** greatly induced the nonradiative relaxation of the excitation energy, resulting in a decrease in fluorescence intensity. With increasing viscosity, on the other hand, the intramolecular rotation of **XTAP**–**Bn** was restricted; thus, the excited state energy was released as a bright NIR fluorescence. More importantly, on the basis of a Förster–Hoffmann equation [21], the plots of log F_765_ against log η with a viscosity change from 1.2 cp to 945 cp exhibited a good linear relationship (*R*^2^ = 0.9959) (Figure 1c), indicating the high sensitivity of **XTAP**–**Bn** to viscosity.

Considering that polarity was an important influencing factor in detecting viscosity, the fluorescence spectra of **XTAP**–**Bn** in some solvents with different polarities were studied. As shown in Figure 1d, the fluorescence intensity of **XTAP**–**Bn** at 765 nm did not show significant changes in different polar solutions compared to that in pure glycerol. These results demonstrated that environmental polarity provided negligible interference when detecting viscosity. Moreover, to broaden the applications in a complex environment, the impacts of environmental pH on the fluorescence response of **XTAP**–**Bn** to viscosity were then investigated (Figure S1). As the pH varied from 3 to 10, the fluorescence intensity changes could be ignored both in low-viscosity (0% glycerol) and high-viscosity (90% glycerol) solutions. This confirmed that **XTAP**–**Bn** was rather stable in relation to pH, and it could be applied to monitor the intracellular viscosity without the interference of pH.

### 2.3. Spectroscopic Response to ClO^−^

**Figure 2:**
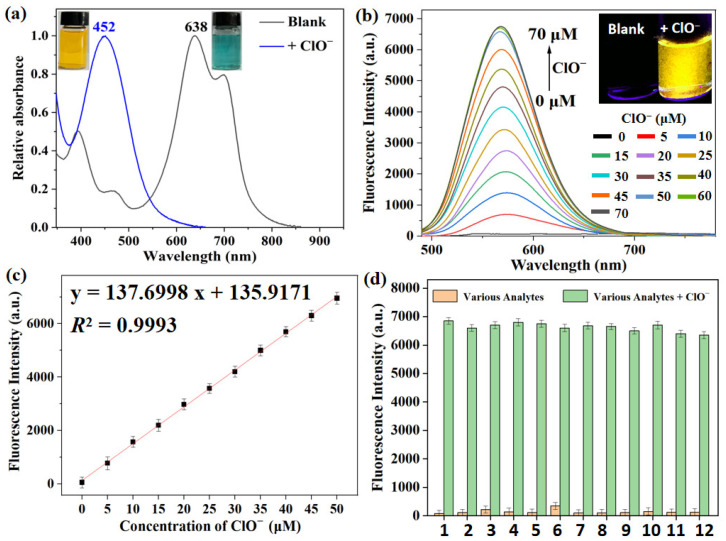
(**a**) The absorption spectra of **XTAP**–**Bn** (5 µM) in PBS buffer without and with ClO^−^ (70 µM). Insert: the corresponding photos taken under sunlight. (**b**) The fluorescence spectra of **XTAP**–**Bn** (5 µM) in PBS buffer after treating with different concentrations of ClO^−^, λ_ex_ = 482 nm. Insert: the corresponding photos taken under 365 nm light irradiation. (**c**) The linear fitting graph of fluorescence intensity at 558 nm with ClO^−^ concentrations from 0 µM to 50 µM. (**d**) The fluorescence intensity of **XTAP**–**Bn** (5 µM) at 558 nm in the presence of various analytes (100 µM) without and with ClO^−^ (50 µM). 1: Blank; 2: ONOO^–^; 3: •OH; 4: ^1^O_2_; 5: H_2_O_2_; 6: Cys; 7: Hcy; 8: GSH; 9: CO_3_^2−^; 10: H_2_PO_4_^−^; 11: S^2−^; 12: SO_3_^2−^. λ_ex_ = 482 nm, error bars are ± SD (n = 3).

Afterward, the spectral response of **XTAP**–**Bn** (5 µM) to ClO^−^ in PBS (10 mM, pH 7.4) was studied. With the addition of ClO^−^ (70 µM), the maximum absorption peak of **XTAP**–**Bn** was significantly blue-shifted from 638 nm to 452 nm, accompanied by a significant change in the color of the solution from dark blue to yellow (Figure 2a). Meanwhile, the fluorescence intensity at 558 nm (F_558_) exhibited an almost 160-fold enhancement (Figure 2b). These phenomena were mainly caused by the ClO−-triggered C=C bond breakage of **XTAP**–**Bn**, which produced the fluorophore **XT**–**CHO** and released a bright-yellow emission. The F_558_ values exhibited an excellent linear relationship (*R*^2^ = 0.9993) with the concentration of ClO^−^ (0–50 µM) (Figure 2c). Based on the 3*σ*/*k* formula, the limit of detection (LOD) was calculated to be 18 nM (Section 3.5), implying that **XTAP**–**Bn** exhibited much higher sensitivity to ClO^−^ than many reported probes (Table S1). As known from the reports, the pathology normal concentration of ClO^–^ in the human body was often set to be within 200 µM [32]. Thus, the feasibility of probe **XTAP**–**Bn** for detecting the higher concentrations of ClO^−^ was investigated. As shown in Figure S2a, when the concentration of **XTAP**–**Bn** was increased to 20 µM, the fluorescence intensity at 558 nm (F_558_) dramatically enhanced with the increase of ClO^−^ concentrations (0–250 µM). Notably, the F_558_ values also exhibited a great linear relationship (*R*^2^ = 0.9951) with the concentration of ClO^−^ (0–200 µM) (Figure S2b). Therefore, probe **XTAP**–**Bn** is promising to achieve quantitative detection of ClO^−^ both at low and high concentrations in living organisms.

To evaluate the selectivity, the fluorescence response of **XTAP**–**Bn** to various potential interferents, including different active oxygen species (ONOO^–^, •OH, ^1^O_2_, H_2_O_2_), some biomolecules (cysteine (Cys), homocysteine (Hcy), glutathione (GSH)), and some common anions (CO_3_^2−^, H_2_PO_4_^−^, S^2−^, SO_3_^2−^) were investigated. We found that ClO^−^ could trigger a significant enhancement of emission at 558 nm, while all other interfering species caused negligible changes (Figure S3). To further ascertain the selective response, an anti-interference test of **XTAP**–**Bn** toward ClO^−^ was carried out in a competitive environment. As shown in Figure 2d, even despite the coexistence with other interfering species, the fluorescence at 558 nm could still be triggered by ClO^−^. The above results suggested that the sensing behaviors of **XTAP**–**Bn** to ClO^−^ were scarcely affected in the presence of other potential interferents.

The response time is an important parameter for evaluating the feasibility and practicability of a probe. In view of this, the time-dependent fluorescence spectra of **XTAP**–**Bn** without and with ClO^−^ were studied (Figure S4). When **XTAP**–**Bn** (5 µM) was excited at 620 nm, its fluorescence intensity remained unchanged within 5 min, indicating that **XTAP**–**Bn** exhibited excellent photostability. However, after treating with ClO^−^ (50 µM), the fluorescence intensity at 558 nm increased rapidly and reached a plateau within 12 s, suggesting that **XTAP**–**Bn** exhibited a much faster response to ClO^−^ than most reported probes (Table S1). Thus, **XTAP**–**Bn** is a promising approach for achieving real-time detection of ClO^−^ in living organisms. To increase practicality, the effect of pH on the response of **XTAP**–**Bn** to ClO^−^ was studied. As shown in Figure S5, the fluorescence intensity of **XTAP**–**Bn** (5 µM) was almost unchanged under different pH conditions, indicating the excellent pH stability of **XTAP**–**Bn**. Upon the addition of ClO^−^ (50 µM), the fluorescence intensity (F_558_) was enhanced significantly in pH, ranging from 4 to 10, suggesting that **XTAP**–**Bn** was capable of ClO^−^ detection in a physiological environment.

### 2.4. Sensing Mechanism

**Figure 3:**
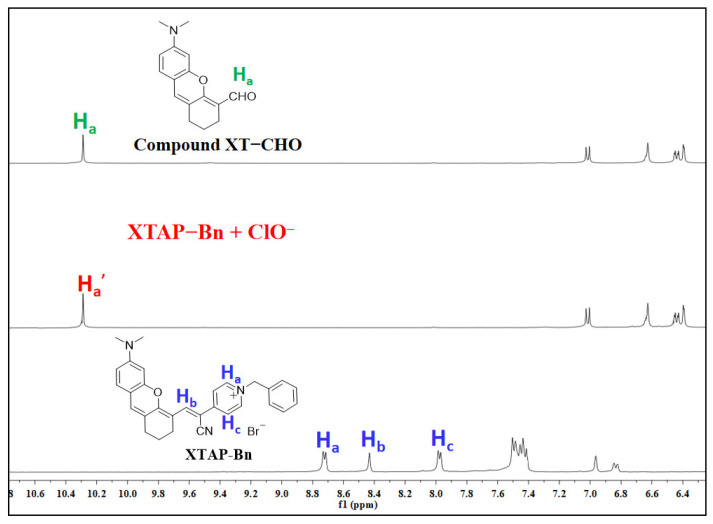
Partial ^1^H NMR spectra of compound **XT**–**CHO**, probe **XTAP**–**Bn**, and the isolated product of **XTAP**–**Bn** + ClO^−^ conducted in DMSO-*d*_6_.

To study the sensing mechanism of **XTAP**–**Bn** in relation to ClO^−^, the reaction product of **XTAP**–**Bn** with ClO^−^ was separated, and its structure was then analyzed by HRMS and ^1^H NMR. As known from the HRMS results (Figure S6), a peak at *m/z* = 446.22267 attributed to **XTAP**–**Bn** (calcd for C_30_H_28_N_3_O (M-Br)^+^ 446.22269) disappeared in the spectrum of **XTAP**–**Bn** + ClO^−^, while a new peak at *m/z* = 256.13332 appeared, and this peak was similar to that of the compound **XT**–**CHO** (calcd for C_16_H_18_NO_2_ (M+H)^+^ 256.13325). Moreover, the ^1^H NMR results showed that the signals of protons on the pyridinium ring at 8.72 (H_a_) and 7.98 (H_c_) ppm, as well as the protons on the acrylonitrile group (H_b_) at 8.43 ppm in **XTAP**–**Bn** all disappeared after the reaction with ClO^−^ (Figure 3). At the same time, a new signal of protons at 10.32 attributed to the aldehyde group (H_a′_) emerged. More importantly, the ^1^H NMR spectra of **XTAP**–**Bn** + ClO^−^ presented the same characteristic peaks as those of the compound **XT**–**CHO**. Finally, the fluorescence spectra of **XTAP**–**Bn** + ClO^−^ were similar to those of the compound **XT**–**CHO** (Figure S7). The above results provided clear evidence that ClO^−^ induced the oxidation breaks of the C=C bond in **XTAP**–**Bn**, thereby producing the fluorophores **XTAP**–**CHO**. This proposed sensing mechanism is illustrated as Scheme 1b.

**Figure 4:**
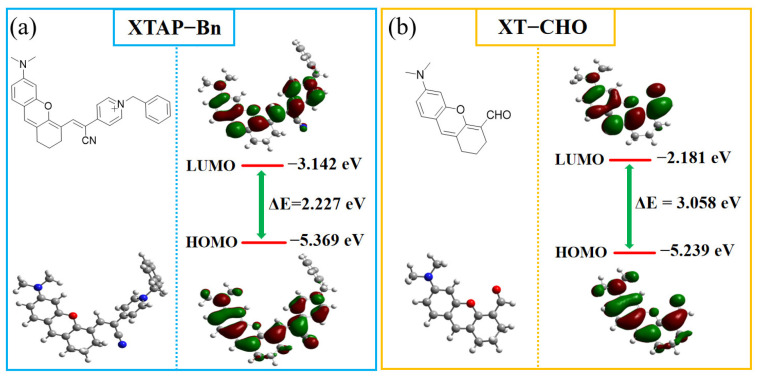
The energy-minimized structures and HOMO/LUMO of probe **XTAP**–**Bn** (**a**) and compound **XT**–**CHO** (**b**) by DFT calculations.

To more fully understand the photophysical properties of **XTAP**–**Bn** in detecting ClO^−^, the electronic structure and the frontier orbital distributions of **XTAP**–**Bn** and the compound **XT**–**CHO** were optimized with density functional theory calculation (DFT) using Gaussian 09 programs with a B3LYP/6-31+G(d) basis set (Figure 4). For **XTAP**–**Bn**, the π electrons on the highest occupied molecular orbital (HOMO) were mainly distributed in the xanthene moiety (electron donor), whereas the electrons on LUMO were primarily arranged in the pyridinium terminal (electron acceptor), which indicates a typical intramolecular charge transfer (ICT) effect from xanthene to pyridinium in the **XTAP**–**Bn** molecule. However, for **XT**–**CHO**, the electrons on LUMO were primarily concentrated in the whole molecule. Moreover, the LUMO–HOMO energy gap (ΔE) of **XT**–**CHO** (3.058 eV) was larger than that of **XTAP**–**Bn** (2.227 eV). The increasing ΔE induced an obvious blue shift of emission wavelength from 765 nm to 558 nm. Notably, these calculated results are highly consistent with the experimental data (Table S4).

### 2.5. Cell Imaging

**Figure 5:**
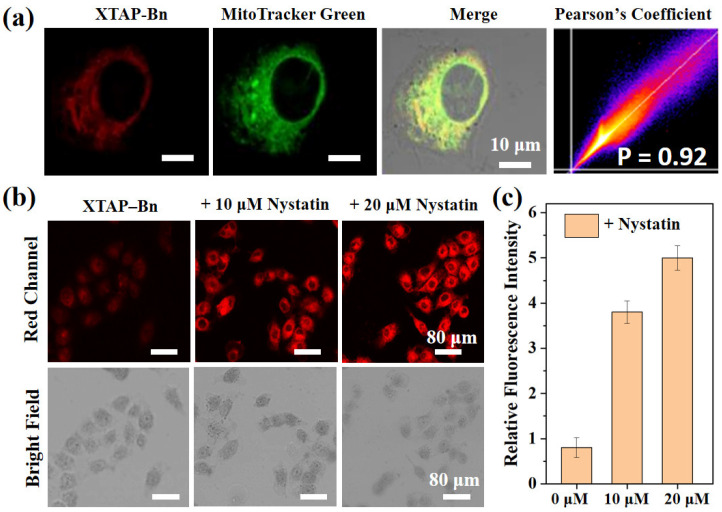
(**a**) Confocal fluorescence image of HeLa cells stained with probe **XTAP**–**Bn** (red channel), commercial dye Mito-Tracker Green (green channel), overlap image, and Pearson correlation coefficient. Green Channel: λ_ex_ = 488 nm; λ_em_ = 500–550 nm. Red Channel: λ_ex_ = 633 nm; λ_em_ = 700–790 nm. (**b**) Confocal imaging of viscosity in living HeLa cells. HeLa cells were pretreated with different concentrations of nystatin (0 µM, 10 µM, and 20 µM) at 37 °C for 45 min and then incubated with **XTAP**–**Bn** (5 µM) at 37 °C for another 30 min. λ_ex_ = 633 nm; λ_em_ = 700–790 nm. (**c**) Relative intensities of cell imaging. Error bars are ± SD (n = 3).

Prior to cell imaging, the cytotoxicity of **XTAP**–**Bn** was accessed via MTT assays in HeLa cells. As shown in Figure S8, even after incubating with 25 µM of **XTAP**–**Bn** for 10 h, the cell survival rate was more than 90%, indicating the low cytotoxicity of **XTAP**–**Bn**. Moreover, owing to the positive charge of the pyridinium moiety, **XTAP**–**Bn** was supposed to be mitochondria-targetable by the electrostatic interaction. In view of this, the mitochondrial targeting ability of **XTAP**–**Bn** was estimated by co-incubating with the commercial Mito-Tracker Green in HeLa cells. According to the results from laser confocal microscopy, HeLa cells co-cultured with **XTAP**–**Bn** exhibited a slightly red emission (Figure 5a), mainly due to the high-viscosity expression in HeLa cells [21]. Moreover, the red channel of **XTAP**–**Bn** and the green channel of Mito-Tracker Green showed a very good overlap, and the Pearson correlation coefficient was 0.92, indicating that **XTAP**–**Bn** can specifically localize in the mitochondria of living cells.

Due to the excellent viscosity sensitivity of **XTAP**–**Bn** in vitro, its application in living cells was then investigated. According to previous reports, nystatin is able to induce the viscosity change of mitochondria and cause cell apoptosis [21,23]. In this case, HeLa cells were co-incubated with 10 µM and 20 µM nystatin at 37 °C for 45 min. After washing three times with PBS, the cells were stained with **XTAP**–**Bn** (5 µM) for another 30 min. As the concentration of co-cultured nystatin increases, the red intracellular fluorescence becomes increasingly bright (Figure 5b,c). These results implied that **XTAP**–**Bn** could be used as an effective tool to monitor viscosity changes in living cells.

**Figure 6:**
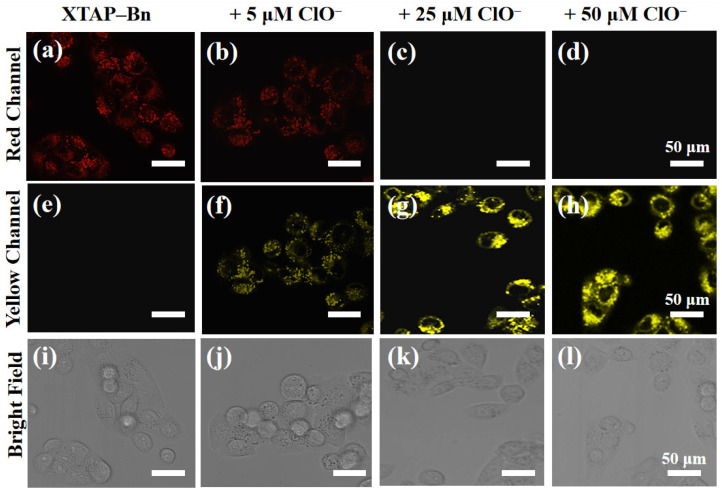
Confocal imaging of ClO^−^ in living HeLa cells. HeLa cells were stained with **XTAP**–**Bn** (5 µM) only (**a**,**e**,**i**), with 5 µM ClO^−^ (**b**,**f**,**j**), with 25 µM ClO^−^ (**c**,**g**,**k**), and with 50 µM ClO^−^ (**d**,**h**,**l**), respectively. Red channel: λ_ex_ = 633 nm; λ_em_ = 700–790 nm. Yellow channel: λ_ex_ = 458 nm, λ_em_ = 520–590 nm.

Afterward, the sensing behavior of **XTAP**–**Bn,** in relation to ClO^−^, was also investigated in living cells. When the HeLa cells were stained only with 5 µM **XTAP**–**Bn** for 30 min, a weak red intracellular fluorescence was observed in the red channel, but no fluorescence was observed in the yellow channel (Figure 6a,e). However, when the cells were cultivated with **XTAP**–**Bn** for 30 min and then treated with different concentrations of ClO^−^ (5 µM, 25 µM, and 50 µM) for another 1 h, the yellow fluorescence in cells significantly brightened (Figure 6f–h). As displayed in Figure S9, the emission intensities in the yellow channel improved steadily with the increase of ClO^−^ concentration. These results implied the concentration-dependent effect of probe **XTAP**–**Bn** toward ClO^−^ in living organisms. The emission intensities in the red channel were dramatically decreased (Figure 6b–d); this was mainly due to the consumption of probe **XTAP**–**Bn** by the released ClO^−^. Therefore, these results confirmed that **XTAP**–**Bn** was able to monitor exogenous ClO^−^ with a yellow fluorescence signal change in living cells.

**Figure 7:**
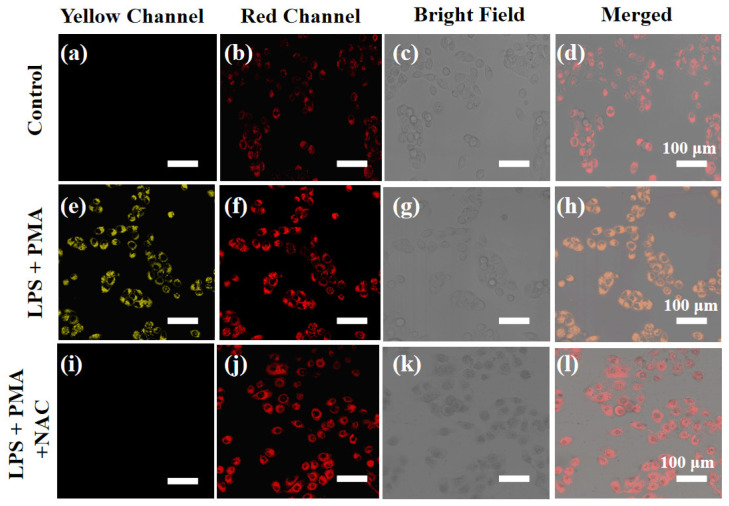
The simultaneous imaging of endogenous ClO^−^ and viscosity in HeLa cells. (**a**–**d**) Cells were stained with **XTAP**–**Bn** (5 µM) for 30 min as a control. (**e**–**h**) Cells were incubated with LPS (300 ng/mL) and PMA (300 ng/mL) for 45 min and then stained with **XTAP**–**Bn** (5 µM) for another 30 min. (**i**–**l**). Cells were incubated with LPS (300 ng/mL) and PMA (300 ng/mL) for 45 min, then NAC (50 µM) for 45 min, and finally stained with **XTAP**–**Bn** (5 µM) for another 30 min. Yellow channel: λ_ex_ = 458 nm, λ_em_ = 520–590 nm; red channel: λ_ex_ = 633 nm; λ_em_ = 700–790 nm.

As known from previous reports, living cells stimulated with lipopolysaccharide (LPS) and N-acetylcysteine (PMA) generate more ClO^−^ [17,18]. It should be noted that LPS can also cause inflammation to increase cell viscosity [21,22]. In view of this, the feasibility of **XTAP**–**Bn** simultaneously detecting ClO^−^ and viscosity was evaluated in HeLa cells after treatment with LPS. As shown in Figure 7, upon treatment of LPS/PMA, the HeLa cells presented significantly increased fluorescence in both yellow and red channels, indicating that more endogenous ClO^−^ was generated, accompanied by an expected increase in viscosity. In addition, the fluorescence enhancement in the yellow channel was inhibited distinctly after incubating with N-Acetyl-L-cysteine (NAC) (a ClO^−^ scavenger), but this had little effect on the fluorescence enhancement of the red channel, further proving that **XTAP**–**Bn** can detect endogenous ClO^−^ and viscosity in cells at the same time.

### 2.6. Zebrafish Imaging

**Figure 8:**
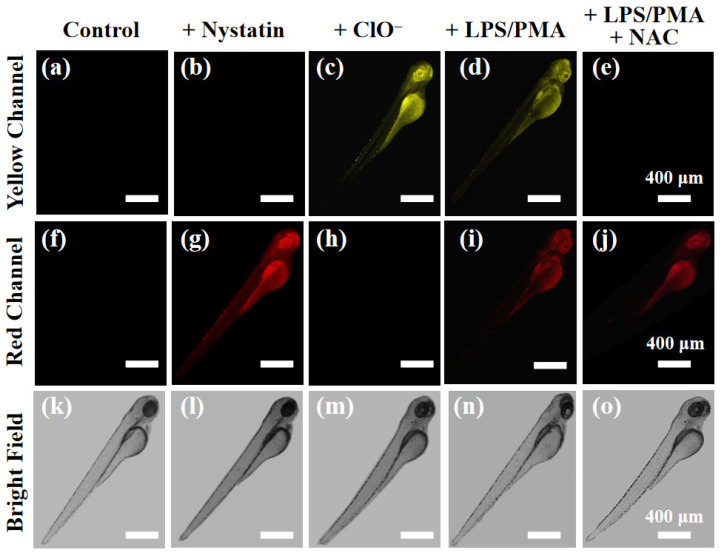
Imaging the dynamic change of ClO^−^ and viscosity levels in living zebrafish. Zebrafish larvae were stained with **XTAP**–**Bn** (5 µM) only as a control (**a**,**f**,**k**), and with 20 µM nystatin (**b**,**g**,**l**), with 50 µM ClO^−^ (**c**,**h**,**m**), with LPS (300 ng/mL) and PMA (300 ng/mL) (**d**,**i**,**n**), with LPS (300 ng/mL), PMA (300 ng/mL) and NAC (50 µM) (**e**,**j**,**o**), respectively. Yellow channel: λ_ex_ = 458 nm, λ_em_ = 520–590 nm; red channel: λ_ex_ = 633 nm; λ_em_ = 700–790 nm.

Encouraged by the excellent performance of cell imaging, the feasibility of **XTAP**–**Bn** visualizing ClO^−^ and viscosity in vivo was studied, and zebrafish larvae were chosen as the vertebrate model. As shown in Figure 8a,f, the zebrafish larvae had non-fluorescence in both the yellow channel and the red channel after treating only with **XTAP**–**Bn** (5 µM). However, upon treatment with nystatin (20 µM) for 2 h, the fluorescence of the red channel was noticeably enhanced (Figure 8g). Subsequently, the ability of **XTAP**–**Bn** to detect ClO^−^ in zebrafish was also investigated. When the zebrafish larvae were stained with **XTAP**–**Bn** (5 µM) for 1 h and then incubated with ClO^−^ (50 µM) for another 1 h, the fluorescence of the yellow channel was markedly improved (Figure 8c). Moreover, after the treatment of LPS/PMA, the zebrafish larvae presented significantly increased fluorescence in both yellow and red channels (Figure 8d,i), indicating that more endogenous ClO^−^ was generated, accompanied by an expected increase in viscosity. While further incubating with NAC, the fluorescence of the yellow channel disappeared due to the scavenging of ClO^−^ (Figure 8e), but the fluorescence of the red channel experienced a negligible change (Figure 8i). These results are highly consistent with those of cell imaging. According to the above results, **XTAP**–**Bn** was capable of simultaneously monitoring the dynamic change of ClO^−^ and viscosity levels in vivo.

## 3. Materials and Methods

### 3.1. Materials and Instruments

Unless otherwise stated, all chemicals were purchased from commercial suppliers and used without further purification. Double-distilled water and chromatographic solvents were used for fluorescence tests. The preparation of various active oxygen species (ROS), some biomolecules, and some common anions were described as follows: (a) ONOO^–^: the stirred solution of NaNO_2_ (0.6 M, 10 mL) and H_2_O_2_ (0.7 M, 10 mL) in deionized H_2_O was added HCl (0.6 M, 10 mL) at 0 °C, immediately followed by the rapid addition of NaOH (1.5 M, 20 mL). Excess hydrogen peroxide was removed by MnO_2_. The concentration of ONOO^–^ was determined by UV analysis with the extinction coefficient at 302 nm, and the solution was stored at −20 °C for use; (b) •OH: to a solution of H_2_O_2_ (10.0 mM, 1.0 mL) in PBS (10 mM, pH 7.4) was added FeSO_4_ solution (10.0 mM, 0.1 mL) at room temperature to get the 1 mM stock solution; (c) ^1^O_2_: the solution of NaMoO_4_ (10 mM) and H_2_O_2_ (10 mM) was prepared in PBS (10 mM, pH = 7.4), respectively, and mixed the equal aliquots of these solutions to afford the 5 mM stock solution of ^1^O_2_; (d) H_2_O_2_, Cys (cysteine), GSH (glutathione), Hcy (homocysteine), and the sodium salts of ClO^−^, CO_3_^2−^, H_2_PO_4_^−^, S^2−^, SO_3_^2−^ were purchased directly from the company, and then diluted with PBS (10 mM, pH = 7.4) to make the 10 mM stock solutions.

For the instruments, a Bruker AV-400 spectrometer was employed to record ^1^H NMR and ^13^C NMR spectra. High-resolution mass spectra (HRMS) were obtained with a Thermo Scientific Q Exactive type mass spectrometer. Melting points were taken with the SGW X-4 instrument (Shanghai, China). Elemental analysis was performed by the Elementar Vario EL instrument (Langenselbold, Germany). Absorption spectra were determined on the TU-1901 UV–vis spectrometer (Beijing, China). Fluorescence spectra were collected by Hitachi F-4500 fluorescence spectrometer (Tokyo, Japan). The fluorescence images of living cells and zebrafish larvae were conducted by Zeiss LSMS880 confocal laser scanning microscope (Jena, Germany).

### 3.2. Synthesis of Compound XTAP

Compound **XT**–**CHO** was first synthesized according to the reported method [33]. After that, compound **XT**–**CHO** (1.02 g, 4.00 mmol) and 4-pyridineacetonitrile (0.54 g, 4.80 mmol) were dissolved in 16 mL ethanol, and then 1 mL piperidine was added into the solution. This mixture was reacted at 70 °C for 10 h under N_2_ atmosphere. After cooling to room temperature, the mixture was concentrated using rotary evaporators, and the remaining solid was purified by column chromatography (DCM/CH_3_OH as eluent, *v/v* = 8:1). The final product **XTAP** was obtained as a dark red solid (0.98 g, 69% yield). m.p. 186.8–188.2 °C. ^1^H NMR (400 MHz, DMSO-*d*_6_): *δ* (ppm) 8.58–8.59 (m, 2H, Pyridyl H), 8.23 (s, 1H, vinyl H), 7.57 (d, *J* = 4.8 Hz, 2H, Pyridyl H), 7.16 (d, *J* = 8.8 Hz, 1H, -ArH), 6.88 (s, 1H, -ArH), 6.71 (s, 1H, -ArH), 6.54–6.57 (m, 1H, -ArH), 3.00 (m, 6H, -(CH_3_)_2_), 2.89 (t, *J* = 5.6 Hz, 2H, -CH_2_), 2.53–2.59 (m, 2H, -CH_2_), 1.75 (t, *J* = 5.6 Hz, 2H, -CH_2_); ^13^C NMR (100 MHz, DMSO-*d*_6_): *δ* (ppm) 155.94, 153.94, 150.17, 150.12, 143.41, 138.06, 128.12, 127.51, 123.22, 118.81, 110.54, 108.71, 107.79, 97.72, 44.58, 29.82, 25.63, 22.02. HRMS (ESI^+^, *m/z*): calcd for C_23_H_21_N_3_O [M+H]^+^, 356.17629; found, 356.17624. Elemental analysis calcd (%) for C_23_H_21_N_3_O: C, 77.72; H, 5.96; O, 4.50; N, 11.82. Found: C, 77.45; H, 5.28; O, 4.88; N, 12.38.

### 3.3. Synthesis of Probe XTAP–Bn

Compound **XTAP** (0.71 g, 2.00 mmol) and (bromomethyl)benzene (0.51 g, 3.00 mmol) were dissolved in 6 mL dry ethanol, and the mixture was then refluxed under the N_2_ atmosphere overnight. After cooling to room temperature, the mixture was concentrated using rotary evaporators, and the obtained residue was then purified using a neutral aluminum oxide column (DCM/CH_3_OH as eluent, *v*/*v* = 10:1). The final product **XTAP**–**Bn** was obtained as a dark purple solid (0.78 g, 74% yield). m.p. 221.6-222.4 °C. ^1^H NMR (400 MHz, DMSO-*d*_6_): *δ* (ppm) 8.72 (d, *J* = 6.4 Hz, 2H, Pyridyl H), 8.43 (s, 1H, vinyl H), 7.98 (d, *J* = 6.8 Hz, 2H, Pyridyl H), 7.41–7.50 (m, 7H, -ArH), 6.96 (s, 1H, -ArH), 6.82–6.84 (m, 1H, -ArH), 5.64 (s, 2H, -CH2), 3.09 (s, 6H, -(CH_3_)_2_), 2.92–2.94 (m, 2H, -CH_2_), 2.66–2.75 (m, 2H, -CH_2_), 1.82–1.84 (m, 2H, -CH_2_); ^13^C NMR (100 MHz, DMSO-*d*_6_): *δ* (ppm) 162.04, 155.07, 153.18, 151.19, 142.74, 139.01, 136.18, 135.05, 129.20, 125.59, 121.52, 119.08, 111.93, 111.30, 110.77, 99.51, 97.12, 67.40, 46.08, 28.53, 25.80, 20.53. HRMS (ESI^+^, *m*/*z*): calcd for C_30_H_28_N_3_O [M-Br]^+^ 446.22269, found 446.22267. Elemental analysis calcd (%) for C_30_H_28_BrN_3_O: C, 68.44; H, 5.36; N, 7.98; O, 3.04. Found: C, 69.10; H, 4.88; N, 7.72; O, 2.86. 

### 3.4. Optical Study

For viscosity response, probe **XTAP**–**Bn** (5 µL, 3 mM in DMSO) was added into 3.0 mL of different viscosity solutions (water/glycerol mixtures with different volume ratios), and the spectra were tested at 20 °C after shaking well. For ClO^−^ detection, the stock solutions of probe **XTAP**–**Bn** (5 µM) were prepared in PBS buffer (10 mM, pH 7.4), and the stock solutions of various ROS (ONOO^–^, •OH, ^1^O_2_, H_2_O_2_), some biomolecules (cysteine, homocysteine, and glutathione) and some common anions (CO_3_^2−^, H_2_PO_4_^−^, S^2−^, and SO_3_^2−^) were prepared as shown in Section 3.3. Unless otherwise stated, all the spectra were recorded at 30 °C for 15 s after treatment with any analyte.

### 3.5. Calculation of the Detection Limit

The detection limit (DL) of probe **XTAP**–**Bn** toward ClO^−^ was calculated by the following equation: DL = 3*σ*/*k*. Where *σ* was the standard deviation of fluorescence intensity for the blank solution, which was measured eight times. *k* represented the slope of the linear calibration plot between the fluorescence intensity and ClO^−^ concentration. According to the linear equation: y = 137.6998x + 135.9171, DL = 18 nM.

### 3.6. Theoretical Calculations

All density functional theory (DFT) calculations were performed with the Gaussian 09 program. The ground state (S0) geometries of probe **XTAP**–**Bn** and compound **XT**–**CHO** were optimized by DFT calculations with B3LYP/6-31G(d) basis set. Moreover, their singlet excited state (S1) geometries were optimized by time-dependent DFT (TDDFT) approaches with the same function program. 

### 3.7. Acytotoxicity Assay

The cytotoxicity was evaluated by MTT assay. HeLa cells were purchased from Wuhan Mingde Biotechnology Co., Ltd., and were incubated in Dulbecco’s modified Eagle medium containing 10% fetal bovine serum, and then the cells were kept at 37 °C under the condition of 5% CO_2_ for 24 h. After that, the cells were incubated with various concentrations of probe **XTAP**–**Bn** (5, 10, 15, 20, and 25 µM) for 10 h. After washing with PBS, 3-(4,5-dimethylthiazol-2-yl)-2,5-diphenyltetrazolium bromide (MTT) was added, and the medium was incubated at 37 °C for 4 h. Finally, the absorbance was read at 490 nm using an ELISA reader (Varioskan Flash). The percentage of cell viability was calculated relative to control wells designated as 100% viable cells.

### 3.8. Living Cell Imaging

All HeLa cells were purchased from Wuhan Mingde Biotechnology Co., Ltd. For the mitochondria targeting study, HeLa cells were firstly cultivated with probe **XTAP**–**Bn** (5 µM) at 37 °C for 30 min in Dulbecco’s Modifed Eagle’s Medium medium containing 10% fetal bovine serum. Afterward, PBS was added for washing three times, and then commercial Mito-Tracker Green and (4 µM) was used to stain the mitochondria. Finally, the fluorescence image was completed using a confocal laser scanning microscope. Green Channel: λ_ex_ = 488 nm; λ_em_ = 500–550 nm. Red Channel: λ_ex_ = 633 nm; λ_em_ = 700–790 nm.

For cellular viscosity imaging, HeLa cells were firstly treated with the different concentrations of nystatin (0 µM, 10 µM, and 20 µM) for 45 min, respectively, and then incubated with **XTAP**–**Bn** (5 µM) at 37 °C for another 30 min. After PBS washing, the image was taken by confocal fluorescence microscopy (λ_ex_ = 633 nm, λ_em_ = 700–790 nm). For cellular ClO^−^ imaging, HeLa cells were firstly stained with **XTAP**–**Bn** (5 µM) for 30 min, and then incubated with different concentrations of NaClO (0 µM, 5 µM, 25 µM, and 50 µM) at 37 °C for 1 h, respectively. After PBS washing, the image was taken by confocal fluorescence microscopy (λ_ex_ = 458 nm, λ_em_ = 520–590 nm). 

For endogenous ClO^−^ and viscosity imaging, HeLa cells were divided into three groups. In a control group, the cells were only stained with probe **XTAP**–**Bn** (5 µM) at 37 °C for 30 min. In the second group, the cells were firstly treated with 300 ng/mL lipopolysaccharide (LPS) and 300 ng/mL N-acetylcysteine (PMA) for 45 min, and then incubated with **XTAP**–**Bn** (5 µM) at 37 °C for another 30 min. In the last group, the cells were treated with 300 ng/mL LPS and 300 ng/mL PMA for 45 min, 50 µM N-acetylcysteine (NAC) for 45 min, and then with **XTAP**–**Bn** (5 µM) at 37 °C for another 30 min. After PBS washing, the image was taken by confocal fluorescence microscopy. Yellow channel: λ_ex_ = 458 nm, λ_em_ = 520–590 nm; red channel: λ_ex_ = 633 nm; λ_em_ = 700–790 nm. 

### 3.9. Zebrafish Imaging

Zebrafish embryos were purchased from Shanghai FishBio Co., Ltd. (Shanghai, China). Larval zebrafish (4 days old) were used for imaging, and they were divided into five groups. In a control group, zebrafish were only cultured with probe **XTAP**–**Bn** (5 µM) at 37 °C for 1 h. In the second group, zebrafish were grown with nystatin (20 µM) for 2 h, and then stained with **XTAP**–**Bn** (5 µM) at 37 °C for another 1 h. In the third group, zebrafish were stained with **XTAP**–**Bn** (5 µM) for 1 h, and then incubated with NaClO (50 µM) at 37 °C for another 1 h. In the fourth group, zebrafish were first treated with 300 ng/mL lipopolysaccharide (LPS) and 300 ng/mL N-acetylcysteine (PMA) for 4 h, and then stained with **XTAP**–**Bn** (5 µM) at 37 °C for another 1 h. In the last group, zebrafish were firstly treated with 300 ng/mL LPS and 300 ng/mL PMA for 4 h, and then cultivated with 50 µM *N*-acetylcysteine (NAC) for 4 h, finally stained with **XTAP**–**Bn** (5 µM) at 37 °C for another 1 h. All zebrafish were washed three times with embryo media, and then transferred to a confocal fluorescence microscopy for imaging. Yellow channel: λ_ex_ = 458 nm, λ_em_ = 520–590 nm; red channel: λ_ex_ = 633 nm; λ_em_ = 700–790 nm.

## 4. Conclusions

In summary, this study presents a novel mitochondria-targeting fluorescent probe, **XTAP**–**Bn**, that can simultaneously detect ClO^−^ and viscosity with off–on yellow fluorescence (558 nm) and NIR fluorescence (765 nm), respectively. The large wavelength gap between these two channels (207 nm) ensured that there was no signal crosstalk during detection, indicating that **XTAP**–**Bn** is obviously superior to previous ClO^−^/viscosity bifunctional probes. Moreover, **XTAP**–**Bn** had the advantages of high selectivity, rapid response, and good water solubility. More importantly, **XTAP**–**Bn** displayed low cytotoxicity and excellent mitochondria localization, and it was successfully employed to monitor the dynamic change in ClO^−^ and viscosity levels in the mitochondria of living cells and zebrafish. This study not only provides a reliable tool for identifying mitochondrial dysfunction but also offers a potential approach for the early diagnosis of mitochondrial-related diseases.

## Data Availability

The data presented in this study are available on request from the corresponding author.

## Supplementary Materials

The following supporting information can be downloaded at: https://www.mdpi.com/article/10.3390/molecules29133059/s1, Table S1: Summary of the recent single detection probes for ClO^−^; Table S2: Summary of the recent single detection probes for viscosity; Table S3: Summary of the recent bifunctional probes for ClO^−^ and viscosity; Table S4: DFT results for **XTAP**–**Bn** and **XT**–**CHO**; Figure S1: pH effect on the fluorescence intensity of probe **XTAP**−**Bn** (5 µM) at 765 nm in water and glycerol (with 10% water). λ_ex_ = 620 nm; Figure S2: (a) Fluorescence spectra of **XTAP**–**Bn** (20 µM) in PBS buffer after treatment with different concentrations of ClO^−^, λ_ex_ = 482 nm. (b) Linear fitting graph of fluorescence intensity at 558 nm with ClO^−^ concentrations from 0 µM to 200 µM; Figure S3: Fluorescence spectra of probe **XTAP**–**Bn** (5 µM) with ClO^−^ (50 µM) and various other species (100 µM) in PBS buffer (10 mM, pH 7.4); Figure S4: The time-dependent experiments of probe **XTAP**–**Bn** (5 µM) without and with ClO^−^ (50 µM); Figure S5: Fluorescence intensity changes of **XTAP**–**Bn** (5 µM) without and with ClO^−^ (50 µM) under different pH conditions; Figure S6: The HRMS data of **XTAP**–**Bn** without and with ClO^−^, as well as compound **XT**–**CHO**; Figure S7: The fluorescence spectra of **XTAP**–**Bn** (5 µM) with ClO^−^ (50 µM) and **XT**−**CHO** (5 µM) in PBS buffer; Figure S8: Viability of HeLa cells after the incubation with different concentrations of probe **XTAP**–**Bn**; Figure S9. Relative intensities of cell imaging; Figure S10: ^1^H NMR (400 MHz, DMSO-*d*_6_) spectrum of **XTAP**; Figure S11: ^13^C NMR (100 MHz, DMSO-*d*_6_) spectrum of **XTAP**; Figure S12: ^1^H NMR (400 MHz, DMSO-*d*_6_) spectrum of **XTAP**–**Bn**; Figure S13: ^13^C NMR (100 MHz, DMSO-*d*_6_) spectrum of **XTAP**–**Bn**; Figure S14: HRMS spectrum of **XTAP**; Figure S15: HRMS spectrum of **XTAP**–**Bn**.

## References

[1] Bazhin A.V. (2020). Mitochondria and cancer. Cancers.

[2] Katherine L.P. (1999). Cytoarchitecture and physical properties of cytoplasm: Volume, viscosity, diffusion, intracellular surface area. Int. Rev. Cytol..

[3] Meszaros A.V., Weidinger A., Dorighello G., Boros M., Redl H., Kozlov A.V. (2016). The impact of inflammatory cytokines on liver damage caused by elevated generation of mitochondrial reactive oxygen species. Free Radic. Biol. Med..

[4] Ramanathan R., Ali A.H., Ibdah J.A. (2022). Mitochondrial dysfunction plays central role in nonalcoholic fatty liver disease. Int. J. Mol. Sci..

[5] Gyasi Y.I., Pang Y.P., Li X.R., Gu J.X., Cheng X.J., Liu J., Xu T., Liu Y. (2020). Biological applications of near infrared fluorescence dye probes in monitoring Alzheimer’s disease. Eur. J. Med. Chem..

[6] Li H., Cao Z., Moore D.R., Jackson P.L., Barnes S., Lambeth J.D., Thannickal V.J., Cheng G. (2012). Microbicidal activity of vascular peroxidase 1 in human plasma via generation of hypochlorous acid. Infect. Immun..

[7] Winterbourn C.C. (2008). Reconciling the chemistry and biology of reactive oxygen species. Nat. Chem. Biol..

[8] Andersen J.K. (2004). Oxidative stress in neurodegeneration: Cause or consequence. Nat. Med..

[9] Ramsey M.R., Sharpless N.E. (2006). ROS as a tumour suppressor. Nat. Cell. Biol..

[10] Yu X., Huang Y., Tao Y., Fan L., Zhang Y. (2024). Mitochondria-targetable small molecule fluorescent probes for the detection of cancer-associated biomarkers: A review. Anal. Chim. Acta.

[11] Fan G., Zhang B., Wang J., Wang N., Qin S., Zhao W., Zhang J. (2024). Accurate construction of NIR probe for visualizing HClO fluctuations in type I, type II diabetes and diabetic liver disease assisted by theoretical calculation. Talanta.

[12] Yuan F., Wang B., Hou J.T., Li J., Shen J., Duan Y., Ren W.X., Wang S. (2023). Demonstrating HOCl as a potential biomarker for liver fibrosis using a highly sensitive fluorescent probe. Sens. Actuators B Chem..

[13] Shao S., Yang T., Han Y. (2023). A TICT-based fluorescent probe for hypochlorous acid and its application to cellular and zebrafish imaging. Sens. Actuators B Chem..

[14] Liang F., Jiang J., Yang X., Zhang G., Zhou J., Han J., Geng Y., Wang Z. (2023). Si-rhodamine fluorescent probe for monitoring of hypochlorous acid in the brains of mice afflicted with neuroinflammation. Chem. Commun..

[15] Fang H., Chen Y., Geng S., Yao S., Guo Z., He W. (2022). Super-resolution imaging of mitochondrial HClO during cell ferroptosis using a near-infrared fluorescent probe. Anal. Chem..

[16] Shangguan L., Wang J., Qian X., Wu Y., Liu Y. (2022). Mitochondria-targeted ratiometric chemdosimeter to detect hypochlorite acid for monitoring the drug-damaged liver and kidney. Anal. Chem..

[17] Kim K.H., Kim S.J., Singha S., Yang Y.J., Park S.K., Ahn K.H. (2021). Ratiometric detection of hypochlorous acid in brain tissues of neuroinflammation and maternal immune activation models with a deep-red/near-infrared emitting probe. ACS Sens..

[18] Xu C., Zhou Y., Li Z., Zhou Y., Liu X., Peng X. (2021). Rational design of AIE-based fluorescent probes for hypochlorite detection in real water samples and live cell imaging. J. Hazard. Mater..

[19] Chen X., Yuan S., Qiao M., Jin X., Chen J., Guo L., Su J., Qu D.H., Zhang Z. (2023). Exploring the depth-dependent microviscosity inside a micelle using butterfly-motion-based fluorescent probes. J. Am. Chem. Soc..

[20] Ge J., Cai W., Niu N., Wen Y., Wu Q., Wang L., Wang D., Tang B.Z., Zhang R. (2023). Viscosity-responsive NIR-II fluorescent probe with aggregation-induced emission features for early diagnosis of liver injury. Biomaterials.

[21] Hong J., Guan X., Chen Y., Tan X., Zhang S., Feng G. (2023). Mitochondrial membrane potential independent near-infrared mitochondrial viscosity probes for real-time tracking mitophagy. Anal. Chem..

[22] Guo Y., Leng H., Chen Q., Su J., Shi W., Xia C., Zhang L., Yan J. (2022). Development of novel near-infrared GFP chromophore-based fluorescent probes for imaging of amyloid-β plaque and viscosity. Sens. Actuators B Chem..

[23] Wu Y., Yin C., Zhang W., Zhang Y., Huo F. (2022). Mitochondrial-targeting near-infrared fluorescent probe for visualizing viscosity in drug-induced cells and a fatty liver mouse model. Anal. Chem..

[24] Xu W., Liu S., Chen Z., Wu F., Cao W., Tian Y., Xiong H. (2022). Bichromatic imaging with hemicyanine fluorophores enables simultaneous visualization of non-alcoholic fatty liver disease and metastatic intestinal cancer. Anal. Chem..

[25] Song H., Zhang W., Zhang Y., Yin C., Huo F. (2022). Viscosity activated NIR fluorescent probe for visualizing mitochondrial viscosity dynamic and fatty liver mice. Chem. Eng. J..

[26] Xu S.L., Guo F.F., Xu Z.H., Wang Y., James T.D. (2023). A hemicyanine-based fluorescent probe for ratiometric detection of ClO^–^ and turn-on detection of viscosity and its imaging application in mitochondria of living cells and zebrafish. Sens. Actuators B Chem..

[27] Chai L., Li Y., Yang H., Wang Y., Huang R., Wei Z., Zhan Z. (2023). pH-triggered fluorescent probe for sensing of hypochlorite and viscosity in live cells and chronic wound diabetic mice. Sens. Actuators B Chem..

[28] Huang X., Luo T., Zhang C., Li J., Jia Z., Chen X., Hu Y., Huang H. (2022). Dual-ratiometric fluorescence probe for viscosity and hypochlorite based on AIEgen with mitochondria-targeting ability. Talanta.

[29] Liang L., Sun Y., Liu C., Jiao X., Shang Y., Zeng X., Zhao L., Zhao J. (2021). Highly selective turn-on fluorescent probe for hypochlorite and viscosity detection. J. Mol. Struct..

[30] Liu T.Z., Wang S., Xu J.R., Miao J.Y., Zhao B.X., Lin Z.M. (2023). FRET-based fluorescent probe with favorable water solubility for simultaneous detection of SO_2_ derivatives and viscosity. Talanta.

[31] Liu Y., Feng S., Gong S., Feng G. (2022). Dual-channel fluorescent probe for detecting viscosity and ONOO^–^ without signal crosstalk in nonalcoholic fatty liver. Anal. Chem..

[32] Chen R., Xing S., Hu T., Chen J., Niu Q., Li T. (2023). Bithiophene-benzothiazole fluorescent sensor for detecting hypochlorite in water, bio-fluids and bioimaging in living cells, plants and zebrafish. Sens. Actuators B Chem..

[33] Chao J.J., Zhang H., Wang Z.Q., Liu Q.R., Mao G.J., Chen D.H., Li C.Y. (2023). A near-infrared fluorescent probe for monitoring abnormal mitochondrial viscosity in cancer and fatty-liver mice model. Anal. Chim. Acta.

