# Supplementary material for: A Mitochondria-Targeting Fluorescent Probe for the Dual Sensing of Hypochlorite and Viscosity without Signal Crosstalk in Living Cells and Zebrafish
Source: Molecules. 2024 Jun 27;29(13):3059. doi: 10.3390/molecules29133059 (PMC11243143; doi:10.3390/molecules29133059)
Supplement: Supplementary file 1 [file molecules-29-03059-s001.zip › molecules-3067022-supplementary.pdf]

Supporting Information

A mitochondria-targeting fluorescent probe for the dual sensing of hypochlorite and viscosity without signal crosstalk in living cells and zebrafish

Chao Gao, Dandan Chen, Lin Zhang, Minglan Ma,
Hu-Wei Liu, Hai-Rong Cui*

Synergy Innovation Centre of Biological Peptide Antidiabetics of Hubei Province, College of Life Science, Wuchang University of Technology, Wuhan, P. R. 430223, P. R. China

* *Correspondence: E-mail:* chr@wut.edu.cn

Table S1 Summary of the recent single detection probes for ClO^-

Probe' Structure	Cell Targeting	Response Time	Detection Limits	Detection Medium	Application	Reference
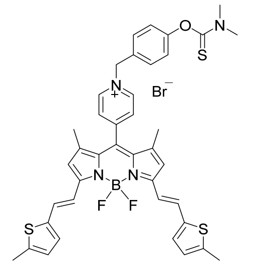	No	5 min	49 nM	CH_3CN/PBS buffer (1:1, v/v)	HeLa cells, Mice	**Ref. 11** *Talanta.* **2024**, *268,* 125298.
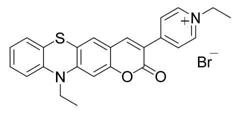	No	10 s	97 nM	PBS buffer with 20% EtOH	PLC cells, HuH-7 cells, HepG2 cells, Mice.	**Ref. 12** *Sens. Actuators B Chem.* **2023**, *378*, 133219.
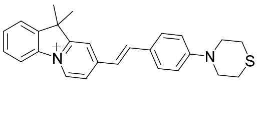	No	40 min	35.2 nM	Tris-HCl buffer (with 1% DMSO)	HeLa cells, zebrafish	**Ref. 13** *Sens. Actuators B Chem.* **2023**, *392*, 134041.
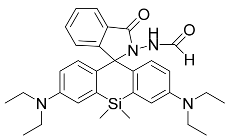	No	35 s	230 nM	PBS buffer with 20% EtOH	HeLa cells, RAW264.7 cells, Mice.	**Ref. 14** *Chem. Commun.* **2023**, *59*, 1357–1360.
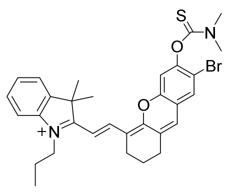	Mitochondria	20 s	89.7 nM	PBS buffer with 10% EtOH	MCF-7 cells, Mice.	**Ref. 15** *Anal. Chem.* **2022**, *94*, 17904−17912.

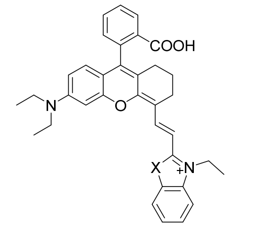	Mitochondria	2.5 min	7.4 nM	PBS buffer	293T cells, Liver tissue slices.	**Ref. 16** *Anal. Chem.* **2022**, *94*, 11881−11888.
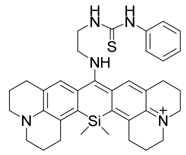	No	5 min	16.1 nM	PBS buffer with 20% DMF	RAW264.7 cells, Mice.	**Ref. 17** *ACS Sens.* **2021**, *6*, 3253–3261.
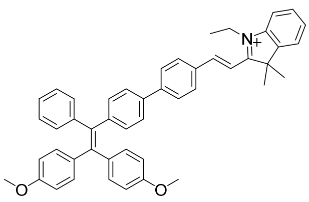	No	5 min	87 nM	PBS buffer with 10% CH_3OH	RAW264.7 cells.	**Ref. 18** *J. Hazard. Mater.* **2021**, *418*, 126243.
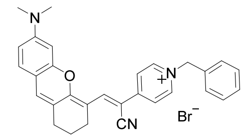	Mitochondria	12 s	18 nM	PBS buffer	HeLa cells, Zebrafish,	**This work**

Table S2 Summary of the recent single detection probes for viscosity

**Probe' Structure**	**Cell Targeting**	**Emission wavelength**	**Application**	**Reference**
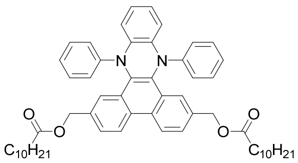	No	600 nm	Vesicle Shell	**Ref. 19** *J. Am. Chem. Soc.* **2023**, *145*, 26494−26503.
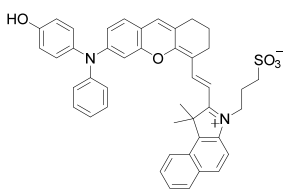	No	911 nm	HeLa cells, Mice	**Ref. 20** *Biomaterials.* **2023**, *300*, 122190.
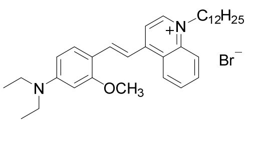	Mitochondria	665 nm	HeLa cells	**Ref. 21** *Anal. Chem.* **2023**, *95*, 5687−5694.
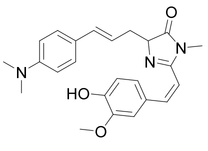	No	702 nm	SH-SY5Y cells, Mice	**Ref. 22** *Sens. Actuators B Chem.* **2022**, *372*, 132648.

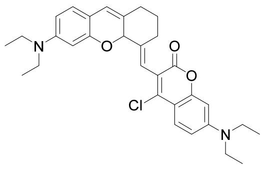	Mitochondrial	722 nm	HeLa cells,<br>Mice	**Ref. 23**<br>*Anal. Chem.*<br>**2022,** *94*,<br>5069−5074.
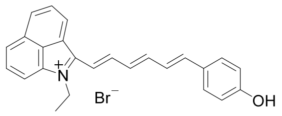	No	795 nm	HeLa cells<br>Mice	**Ref. 24**<br>*Anal. Chem.*<br>**2022**, *94*,<br>13556−13565.
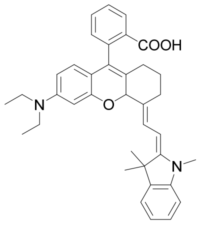	Mitochondria	739 nm	HL-7702 cells,<br>Mice	**Ref. 25**<br>*Chem. Eng. J.*<br>**2022**, *445*, 136448.
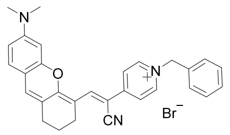	Mitochondria	765 nm	HeLa cells,<br>Zebrafish	**This work**

Table S3 Summary of the recent bifunctional probes for ClO^- and viscosity

Probe' Structure	Cell Targeting	For ClO^- (λ_{em})	For Viscosity (λ_{em})	Emission wavelength difference ($\Delta\lambda$ nm)	Application	Reference
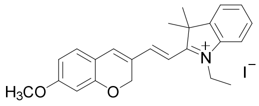	Mitochondria	493 nm	628 nm	**135 nm**	C6 cells,<br>Zebrafish	**Ref. 26**<br>*Sens. Actuators B Chem.*<br>**2023**, *383*,<br>133510.
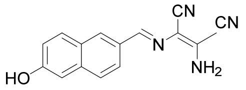	No	520 nm	620 nm	**100 nm**	HeLa cells,<br>HepG2 cells,<br>Mice	**Ref. 27**<br>*Sens. Actuators B Chem.*<br>**2023**, *393*,<br>134345.
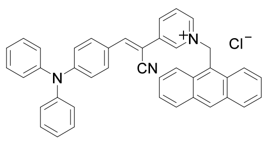	Mitochondria	414 nm	600 nm	**186 nm**	HeLa cells,	**Ref. 28**<br>*Talanta.*<br>**2022**, *241*,<br>123235.
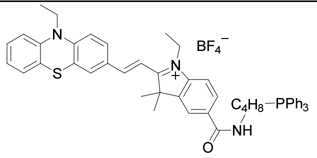	No	510 nm	600 nm	**90 nm**	INS-1 cells	**Ref. 29**<br>*J. Mol. Struct.*<br>**2021**, *1227*,<br>129523.
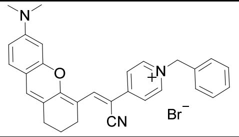	Mitochondria	558 nm	765 nm	**207 nm**	HeLa cells,<br>Zebrafish	**This work**

Table S4: DFT results for XTAP−Bn and XT−CHO[a]

Compounds	HOMO[b] (eV)	LUMO[b] (eV)	Eg[b] (eV)	λ_{em}[b]/nm	λ_{em}[c]/nm
XTAP−Bn	-5.369	-3.142	2.227	746 nm	765 nm
XT−CHO	- 5.239	- 2.181	3.058	574 nm	558 nm

[a] DFT calculation is performed with Gaussian 09 programs at B3LYP/6-31G(d) basis set.

[b] The calculation result was obtained using H_2O as the solvent.

[c] Emission maximum (λ_{em}) measured in PBS buffer.

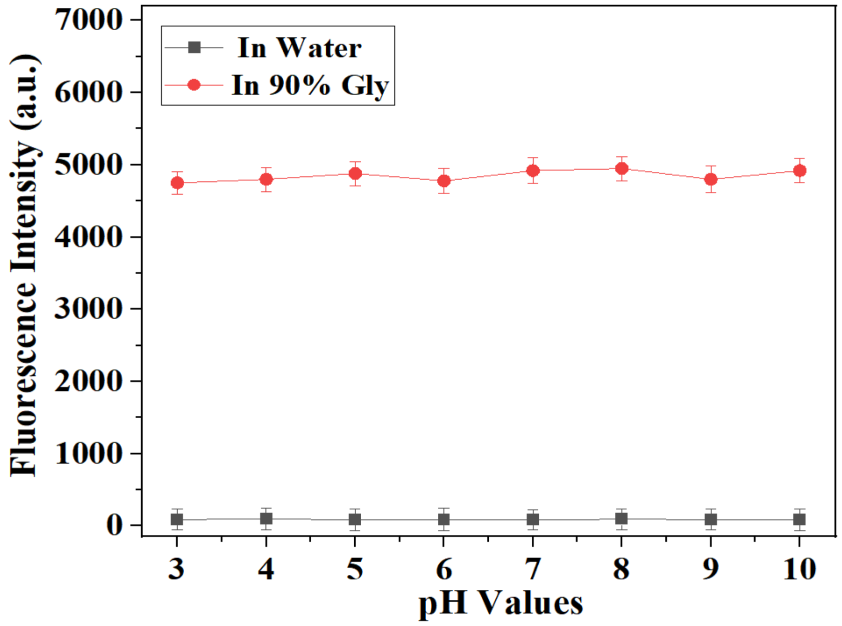

Figure S1. pH effect on the fluorescence intensity of probe **XTAP−Bn** (5 μM) at 765 nm in water and glycerol (with 10 % water). λ_{ex} = 620 nm.

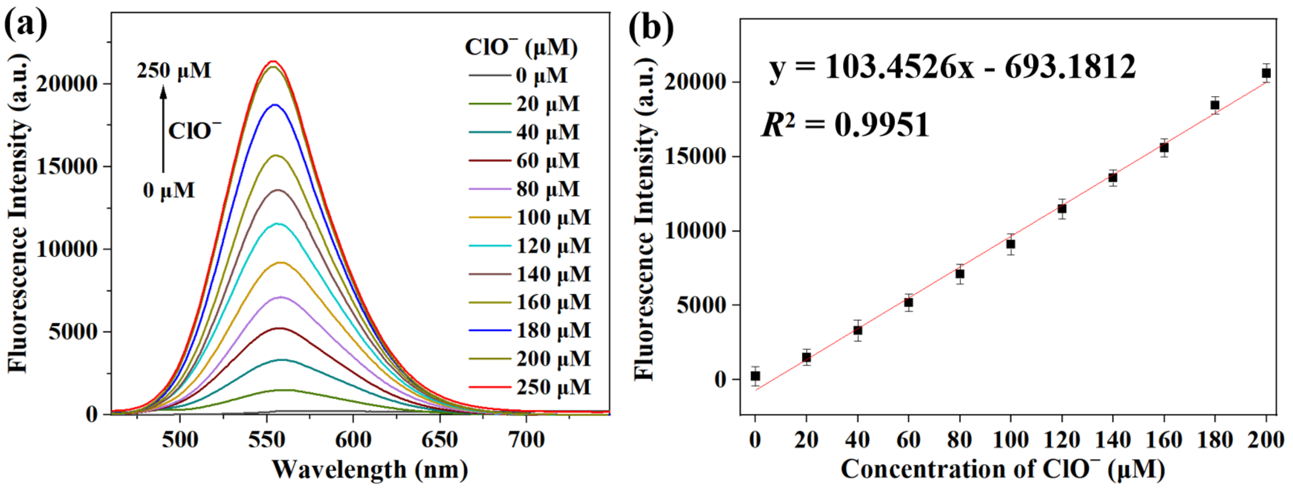

Figure S2. (a) Fluorescence spectra of **XTAP−Bn** (20 μM) in PBS buffer after treating with different concentration of ClO^-, λ_{ex} = 482 nm. (b) Linear fitting graph of fluorescence intensity at 558 nm with ClO^- concentrations from 0 μM to 200 μM.

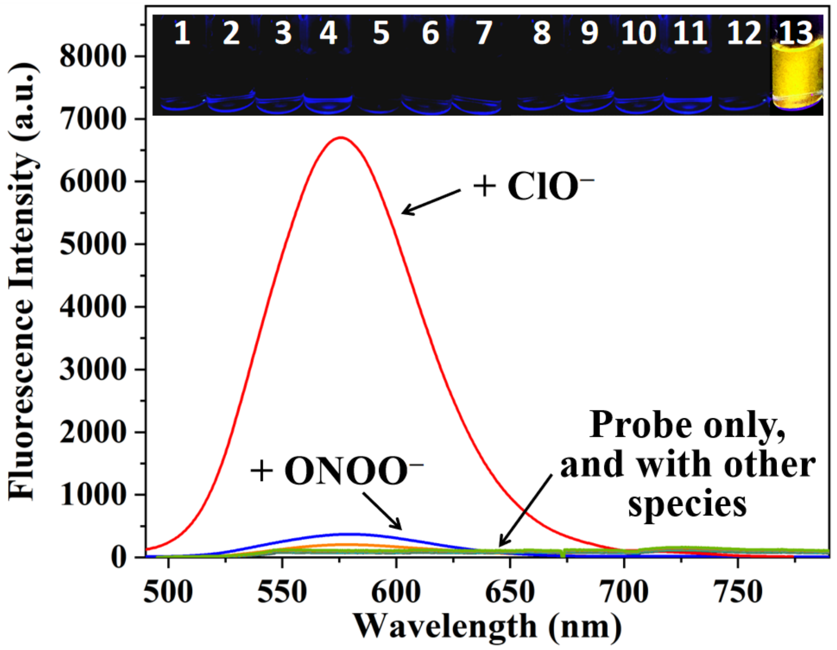

Figure S3. Fluorescence spectra of probe **XTAP−Bn** (5 μM) with ClO^- (50 μM) and various other species (100 μM) in PBS buffer (10 mM, pH 7.4). Insert: the corresponding photos taken under 365 nm light irradiation. 1: Blank; 2: $ONOO^-$; 3: •OH; 4: $^1O_2$; 5: H_2O_2; 6: Cys; 7: Hcy; 8: GSH; 9: CO_3^{2-}; 10: $H_2PO_4^-$; 11: S^{2-}; 12: SO_3^{2}; 13: ClO^-. λ_{ex} = 482 nm.

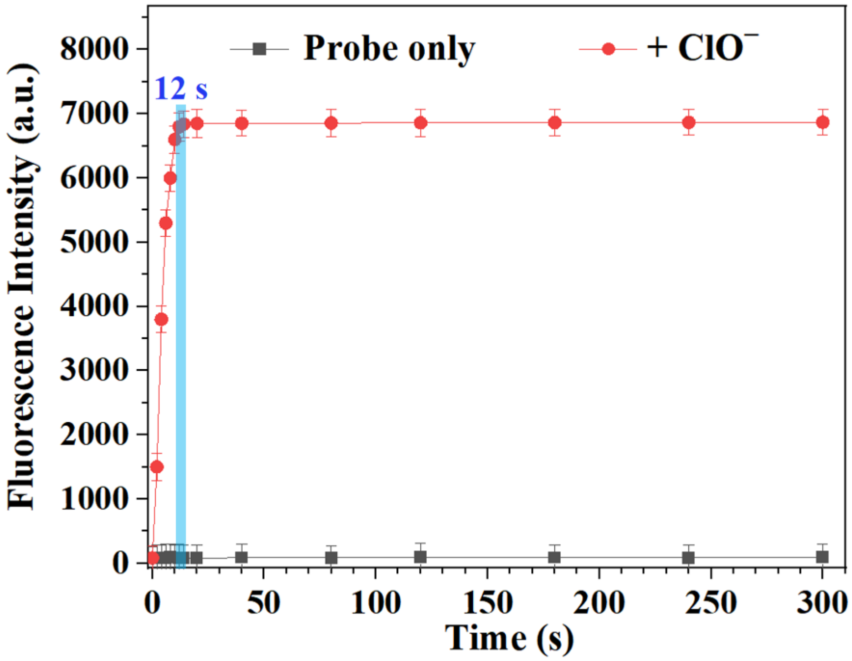

Figure S4. The time-dependent experiments of probe **XTAP−Bn** (5 μM) without and with ClO^- (50 μM). For probe **XTAP−Bn** only: λ_{ex} = 620 nm; λ_{em} =765 nm; For **XTAP−Bn** with ClO^-: λ_{ex} = 482 nm; λ_{em} = 558 nm. Error bars are ± SD (n = 3).

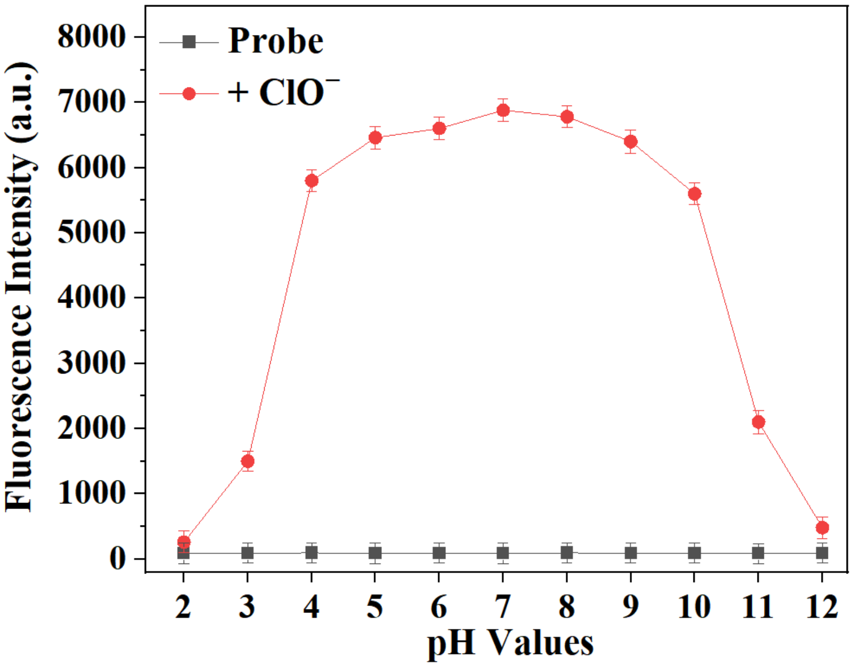

Figure S5. Fluorescence intensity changes of **XTAP−Bn** (5 μM) without and with ClO^- (50 μM) under different pH conditions. For probe **XTAP−Bn** only: λ_{ex} = 620 nm; λ_{em} =765 nm; For **XTAP−Bn** with ClO^-: λ_{ex} = 482 nm; λ_{em} = 558 nm. Error bars are ± SD (n = 3).

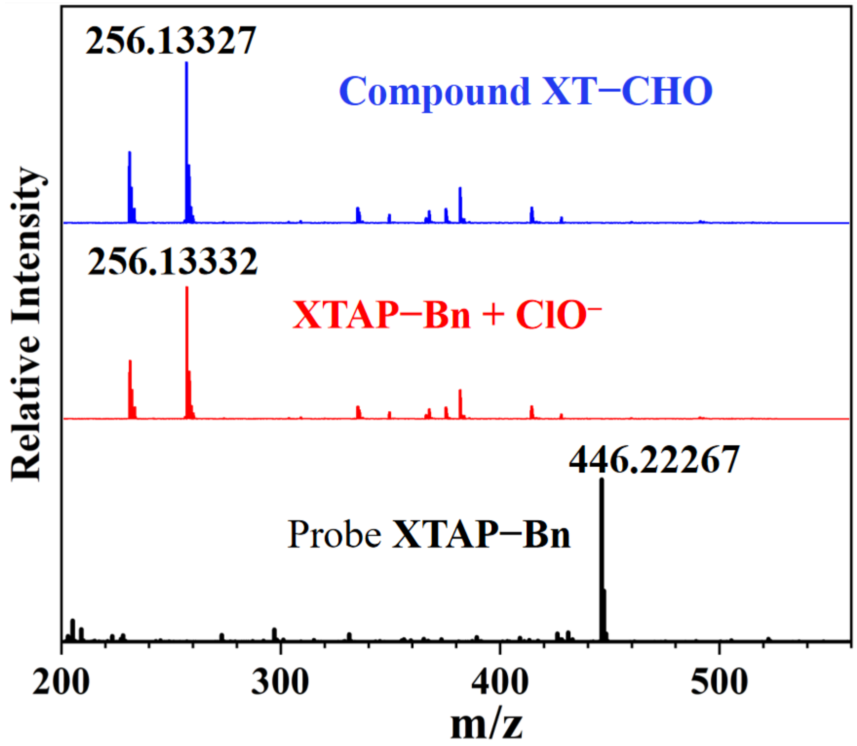

Figure S6. The HRMS data of **XTAP−Bn** without and with ClO^-, as well as compound **XT−CHO**.

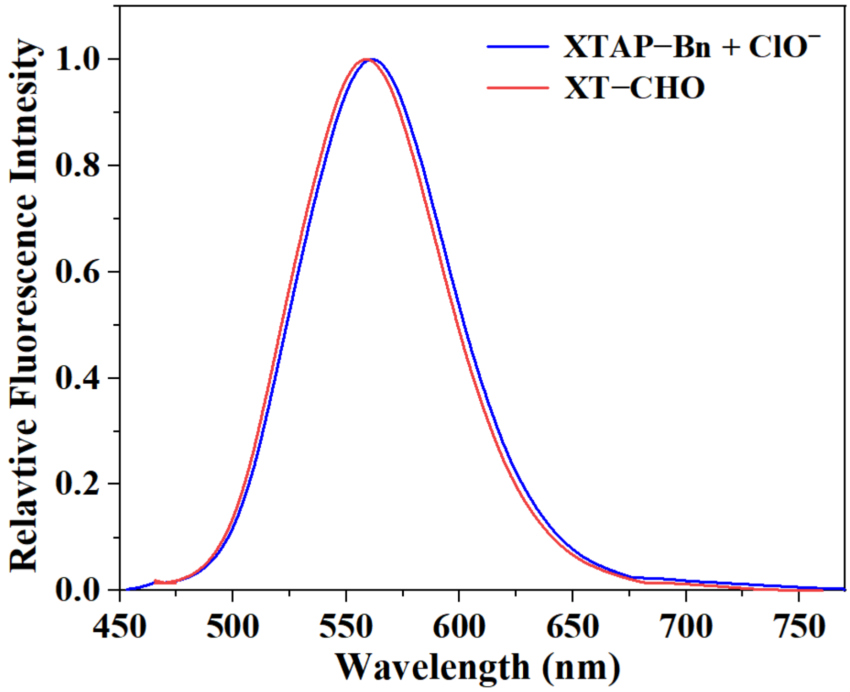

Figure S7. The fluorescence spectra of **XTAP−Bn** (5 μM) with ClO^- (50 μM) and **XT−CHO** (5 μM) in PBS buffer.

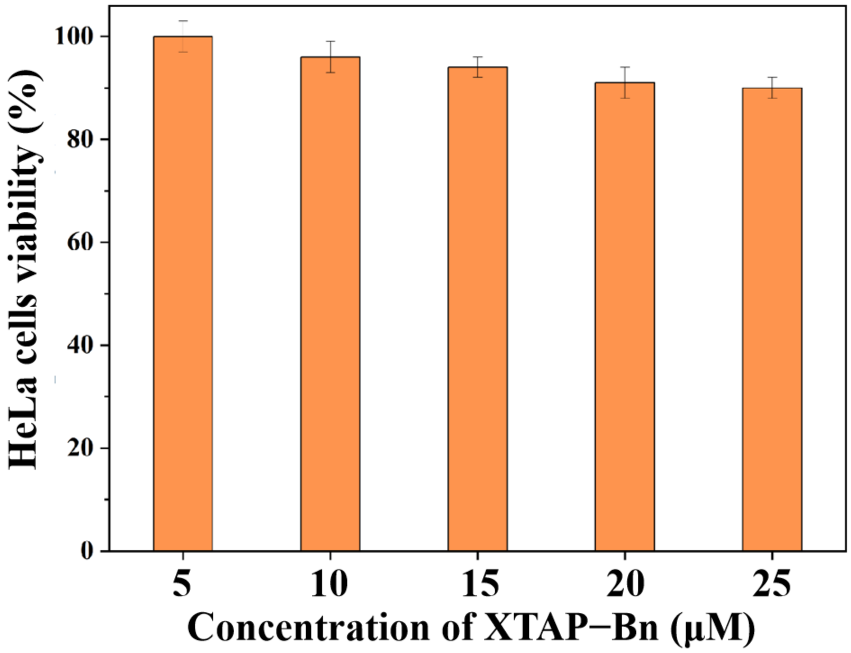

Figure S8. Viability of HeLa cells after the incubation with different concentrations of probe **XTAP−Bn**.

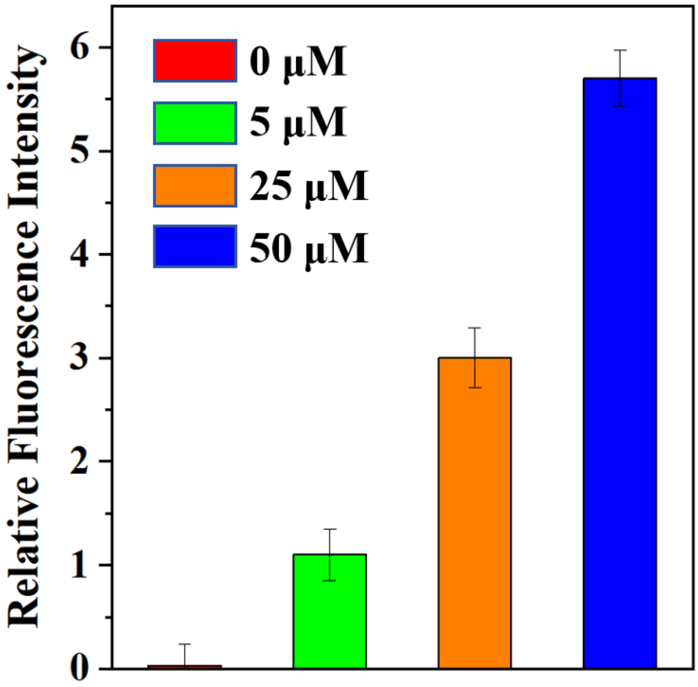

Figure S9. Relative intensities of cell imaging. HeLa cells were firstly stained with **XTAP−Bn** (5 μM) for 30 min, and then incubated with different concentration of NaClO (0 μM, 5 μM, 25 μM, and 50 μM) at 37 °C for 1 h, respectively. λ_{ex} = 458 nm, λ_{em} = 520 nm−590 nm. Error bars are ± SD (n = 3).

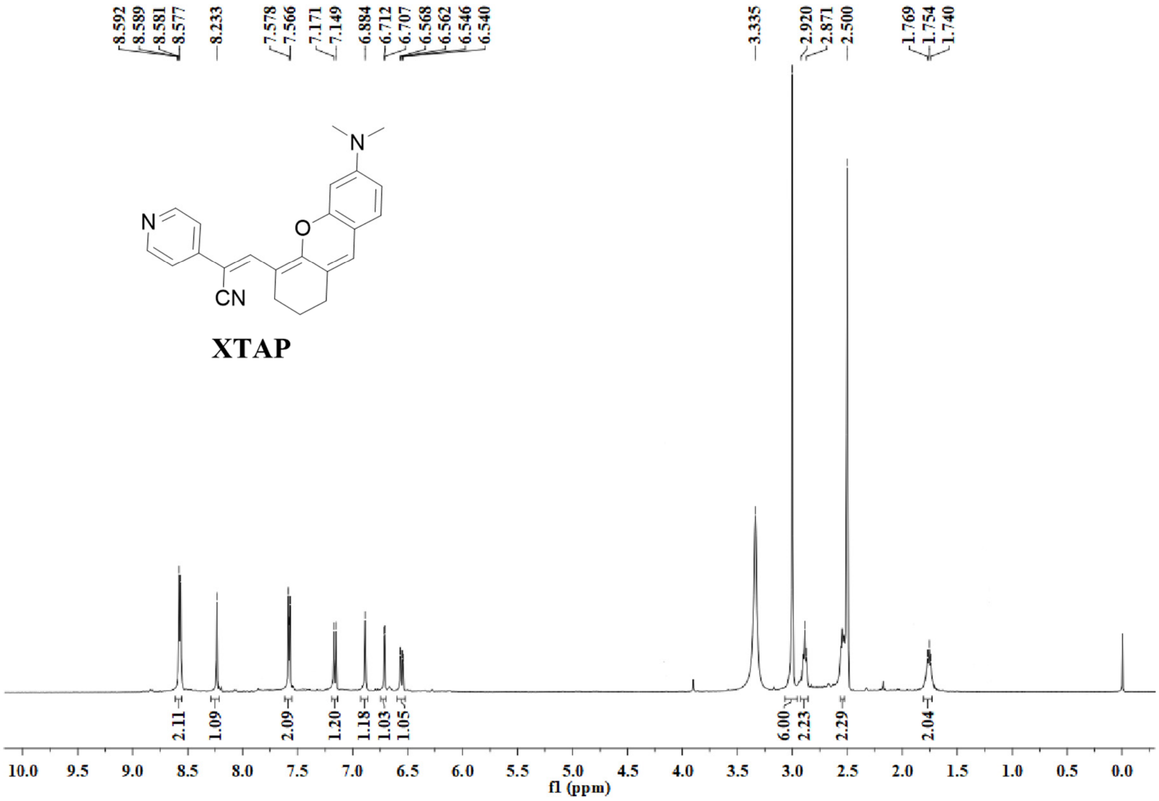

Figure S10. ^{1}H NMR (400 MHz, DMSO-d_6) spectrum of **XTAP.**

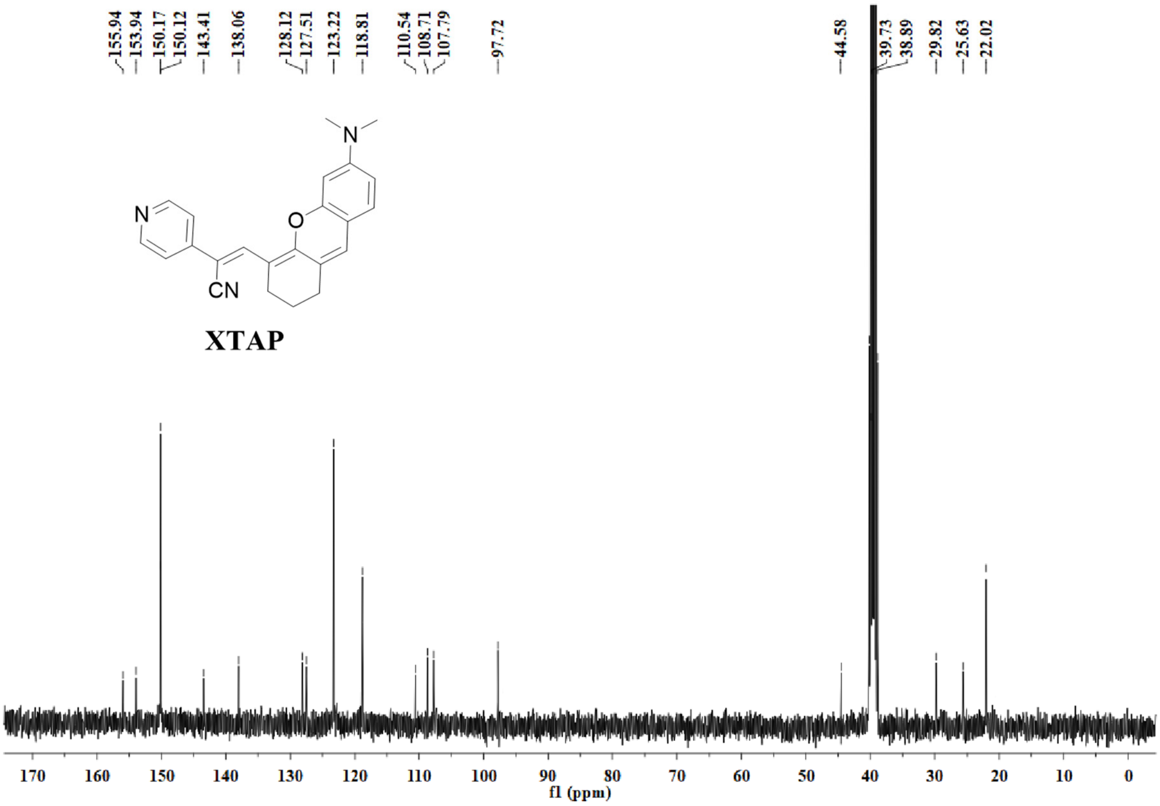

Figure S11. ^{13}C NMR (100 MHz, DMSO-d_6) spectrum of **XTAP.**

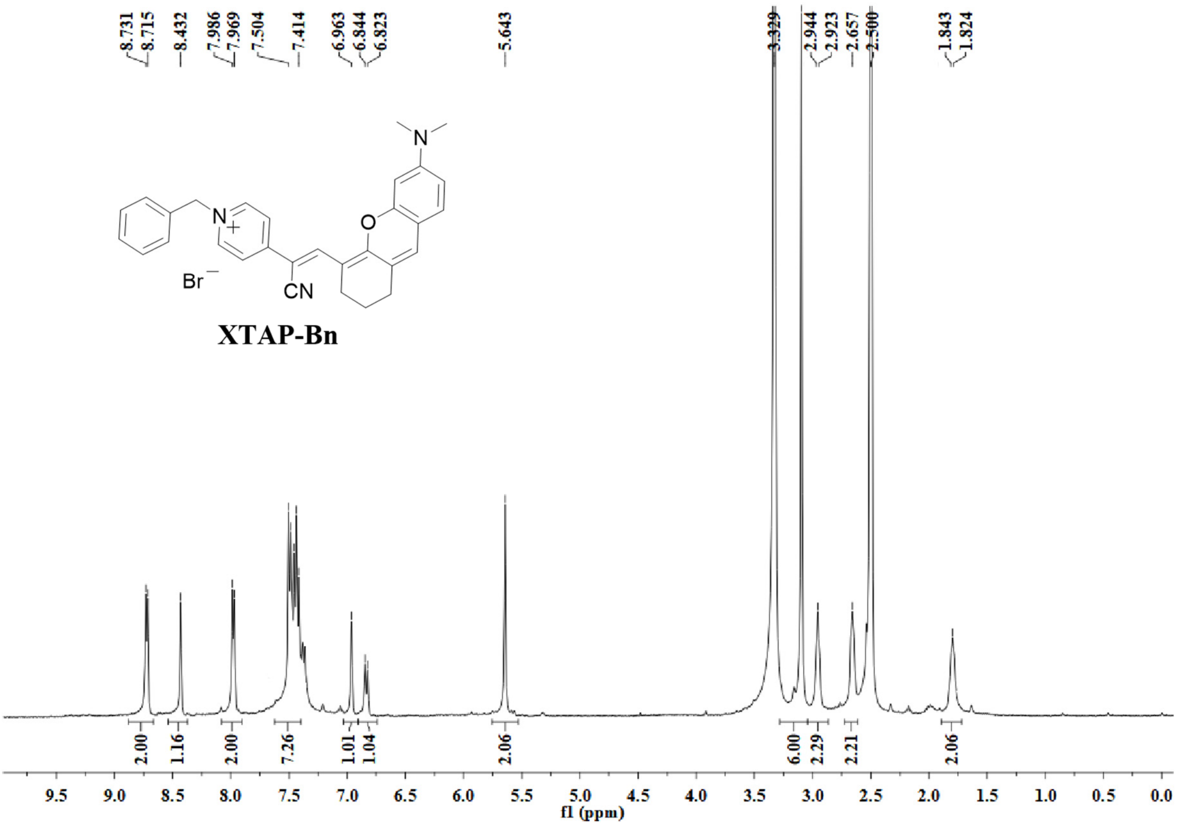

Figure S12. ^{1}H NMR (400 MHz, DMSO-d_6) spectrum of **XTAP−Bn.**

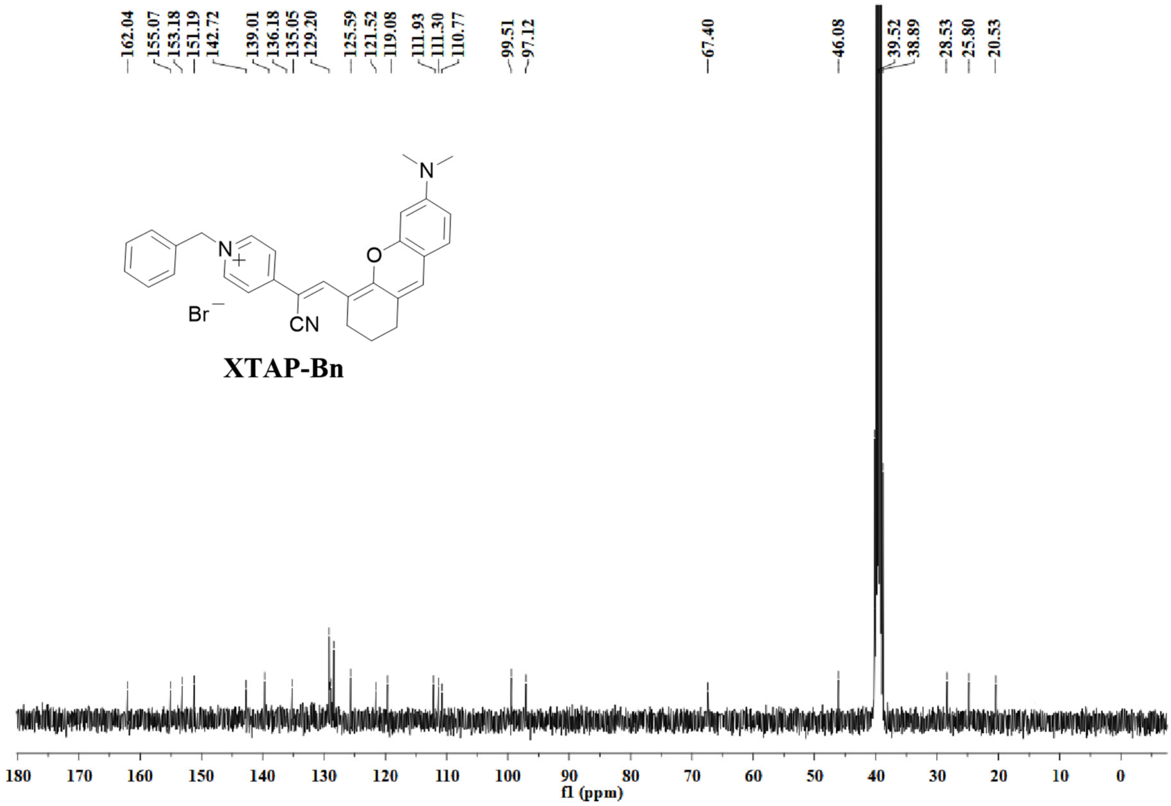

Figure S13. ^{13}C NMR (100 MHz, DMSO-d_6) spectrum of **XTAP−Bn.**

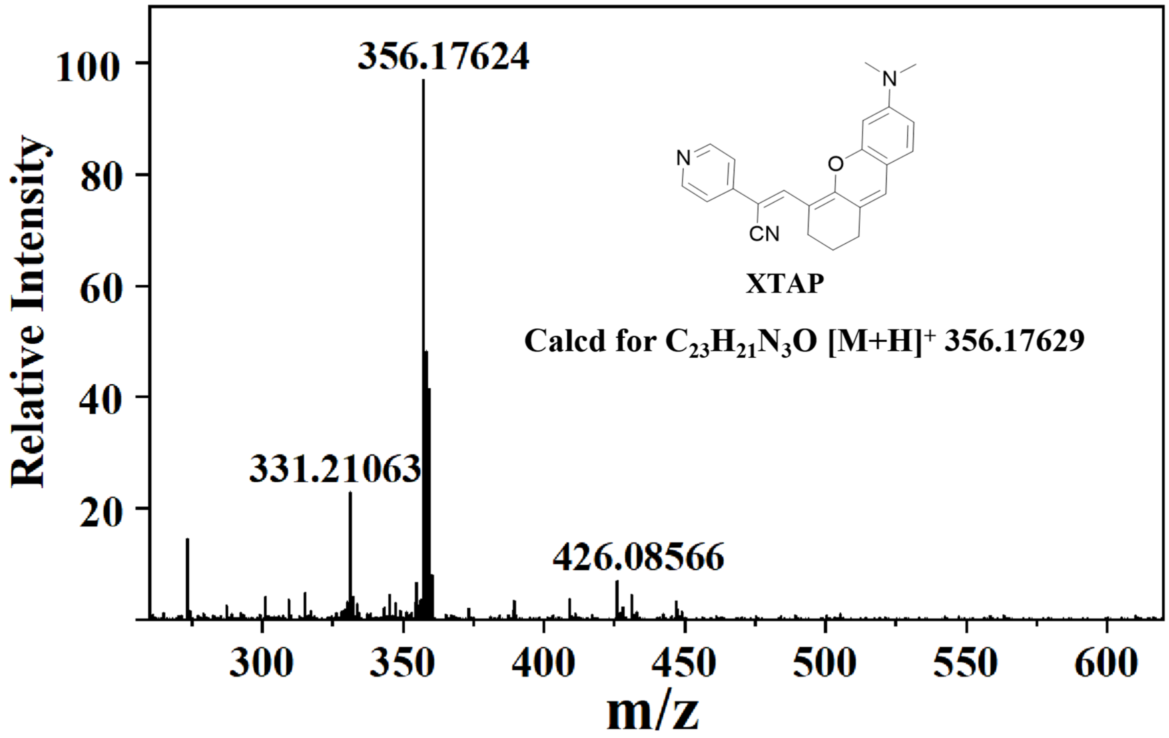

Figure S14. HRMS spectrum of **XTAP.**

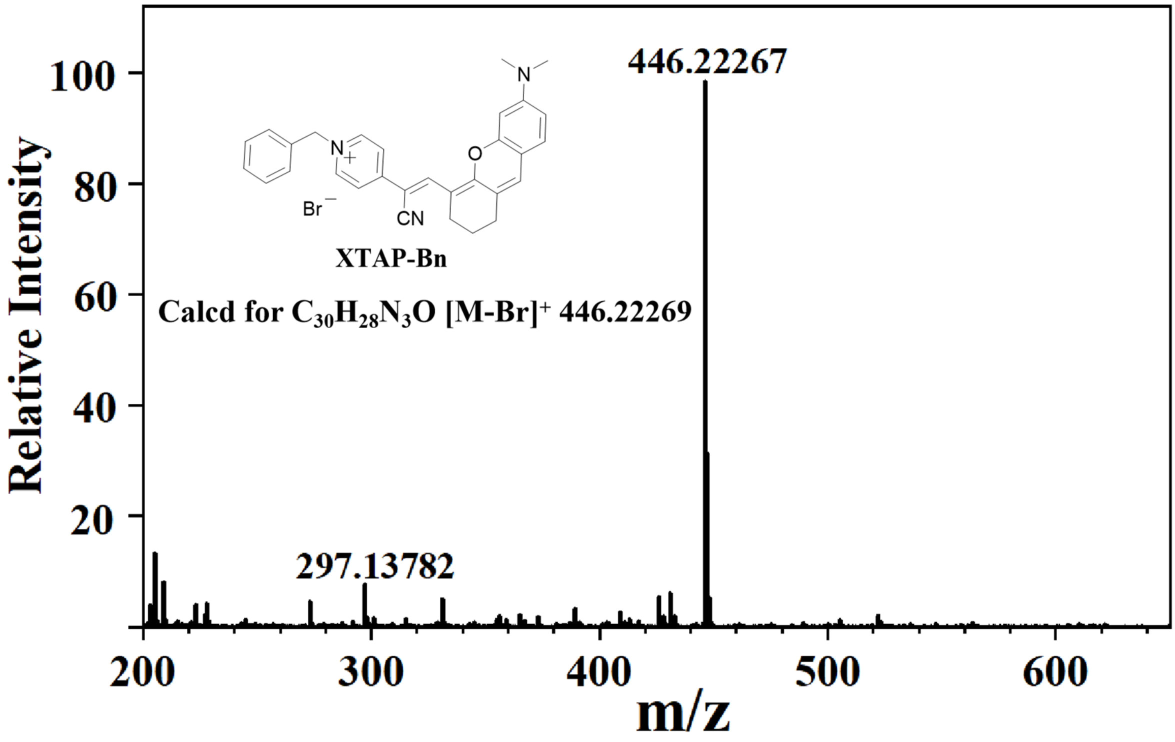

Figure S15. HRMS spectrum of **XTAP−Bn.**
